# Functional and structural responses of plankton communities toward consecutive experimental heatwaves in Mediterranean coastal waters

**DOI:** 10.1038/s41598-023-35311-4

**Published:** 2023-05-17

**Authors:** Tanguy Soulié, Francesca Vidussi, Sébastien Mas, Behzad Mostajir

**Affiliations:** 1 MARBEC (MARine Biodiversity, Exploitation and Conservation), Univ Montpellier, CNRS, Ifremer, IRD, Montpellier, France; 2 MEDIMEER (MEDIterranean Platform for Marine Ecosystems Experimental Research), OSU OREME, CNRS, Univ Montpellier, IRD, INRAE, Sète, France

**Keywords:** Ecology, Ecology, Environmental sciences, Ocean sciences

## Abstract

The frequency of marine heatwaves (HWs) is projected to increase in the Mediterranean Sea over the next decades. An in situ mesocosm experiment was performed in a Mediterranean lagoon for 33 days. Three mesocosms were used as controls following the natural temperature of the lagoon. In three others, two HWs of + 5 °C compared to the controls were applied from experimental day (d) 1 to d5 (HW1) and from d11 to d15 (HW2). High-frequency data of oxygen, chlorophyll-*a* (chl-*a*), temperature, salinity and light from sensors immersed in all mesocosms were used to calculate gross primary production (GPP), respiration (R) and phytoplankton growth (µ) and loss (L) rates. Nutrients and phytoplankton community structure from pigments were also analyzed. HW1 significantly increased GPP, R, chl-*a*, µ and L by 7 to 38%. HW2 shifted the system toward heterotrophy by only enhancing R. Thus, the effects of the first HW resulted in the attenuation of those of a second HW on phytoplankton processes, but not on community respiration, which was strongly regulated by temperature. In addition, natural phytoplankton succession from diatoms to haptophytes was altered by both HWs as cyanobacteria and chlorophytes were favored at the expense of haptophytes. These results indicate that HWs have pronounced effects on Mediterranean plankton communities.

## Introduction

Marine heatwaves (HWs) are extreme short-lived warming events lasting from several days to months^1^. They are projected to increase in frequency and intensity in most oceans worldwide in the coming decades due to global climate change^2,3^. They are expected to have profound economic and ecological consequences^4^, particularly in Mediterranean coastal waters, which are among the area most sensitive toward their intensification^5–8^. Therefore, studying the effects of HWs on fundamental plankton functions within ecosystems is essential because plankton communities play crucial roles in aquatic ecosystems^9^.

Phytoplankton produces oxygen through its photosynthesis, this oxygen production can be referred to as gross primary production (GPP), whereas all plankton consumes oxygen via aerobic respiration (R). Therefore, the balance between GPP and R provides a metabolic index for aquatic systems, representing their capacity to either act as net consumers (GPP < R) or net producers of oxygen (GPP > R^10,11^). This balance is partially related to phytoplankton growth (µ) and loss (L) rates, which themselves give a trophic index of the system related to the fitness of phytoplankton and of its factors of loss, such as predation and viral lysis^12–14^. Even if all phytoplankton take part in primary production, phytoplankton functional groups can differ in their roles within food webs and biogeochemical cycles. This means that the phytoplankton community structure is closely related to both the balance between GPP and R and the balance between µ and L^15^. Therefore, assessing the response of these indices and of phytoplankton community structure is critical to deepen our understanding of the consequences of HWs on Mediterranean coastal ecosystem functioning.

The effects of natural HWs on plankton communities and processes have been studied in several systems. Observations reported important effects of HWs on phyto- and zooplankton communities in various regions^16–20^, and suggested that HWs tend to boost phytoplankton biomass at high latitudes in the open ocean with decreases at mid- and tropical latitudes^21–23^. Accordingly, a physical–biogeochemical model and a marine HW framework have predicted positive effects of HWs on phytoplankton biomass and growth under nutrient-replete conditions, and the opposite under nutrient-depleted conditions^24,25^. These predictions have been confirmed in the P-depleted Mediterranean Sea, where phytoplankton production has been negatively affected by an increase in the intensity, frequency, and duration of HWs since 1985^26^. However, responses can be substantially more complex in coastal areas, and depend notably on local coastal processes, such as allochthonous nutrient inputs from land and exchanges with the benthic compartment^27^. Indeed, indirect and/or cascading effects of warming in coastal systems have frequently been highlighted during experimental studies, depending on the environmental conditions and plankton community structure at the beginning of the study. In Mediterranean coastal waters, warming was shown to either promote or depress phytoplankton growth and biomass depending on nutrient status; change the structure and interactions among phytoplankton, zooplankton, bacterioplankton, and virioplankton communities; and shift metabolic status toward either autotrophy or heterotrophy^28–33^. In addition to the effects of warming that occur directly during HWs, changes in plankton key functions and community structure occurring after the end of the HW have been reported^32,34,35^. This means the responses of aquatic ecosystems to HWs are more complex than a simple response to warming. Although most studies have focused on one experimental HW^32,36,37^, the consequences of consecutive HWs are largely unknown and so almost unpredictable, and it might be difficult to infer the responses to consecutive HWs from studies investigating only one HW. Indeed, the exposure to a first HW may impact the responses to a second HW because it may deeply modify functional processes, community composition and interactions among organisms, which in turn can determine the responses to the second HW.

To assess the effects of consecutive HWs on Mediterranean coastal plankton communities, a one-month in situ mesocosm experiment was performed from April 28 to May 31, 2022 in Thau lagoon. This shallow productive lagoon is located on the French coast of the northwestern Mediterranean Sea^38^, and high-frequency water temperature monitoring has shown that consecutive HWs have already occurred in the lagoon in the past^39^. During the experiment, two consecutive five-day HWs were simulated in triplicate mesocosms by heating the water at + 5 °C compared to controls. The responses of the metabolic and the trophic indices were estimated from continuous high-frequency measurements. Meanwhile, manual sampling was used to assess the chemical conditions and phytoplankton community structure.

## Results

### Physico-chemical and biological conditions from high-frequency measurements

**Figure 1:**
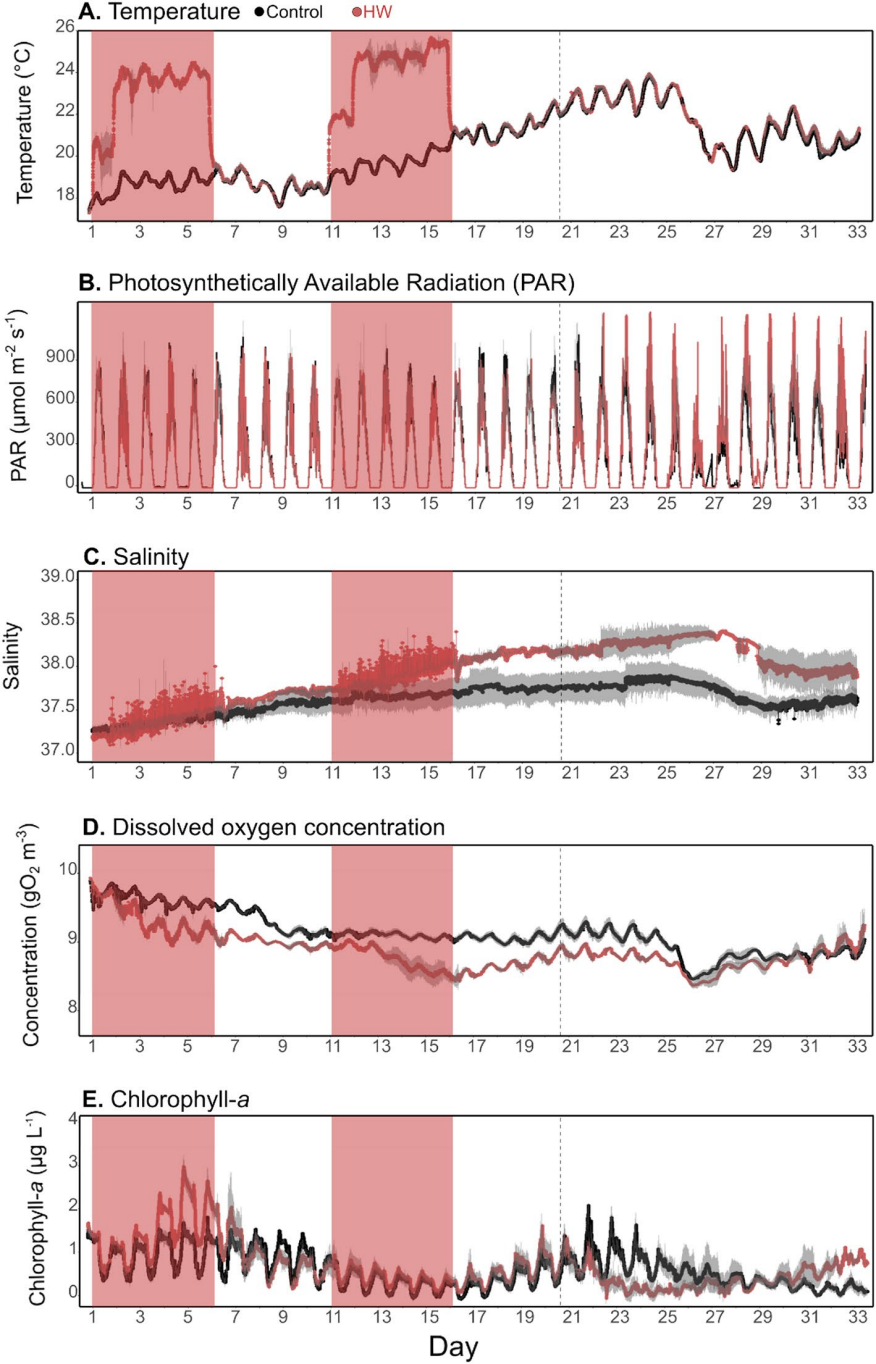
High-frequency sensor data obtained during the experiment. Mean water temperature (**A**), photosynthetically available radiation (PAR, **B**), salinity (**C**), dissolved oxygen concentration (**D**), and chlorophyll-*a* (**E**) obtained from high-frequency sensors immersed at 1 m depth in all the mesocosms over the course of the experiment in the control (black) and HW (red) treatments. The red shaded areas represent the HW1 and HW2 periods. The vertical dashed line indicates the end of the Post-HW2 period (d20). The grey shaded area represents the standard deviation (n = 3) for the control treatment and the range of observations (n = 2) for the HW treatment.

The water temperature ranged from 17.8 ± 0.01 (d1) to 23.14 ± 0.01 °C (d25) in the control treatment (Fig. 1A). It increased until d24, before decreasing and stabilizing at approximately 21 °C until the end of the experiment. In the HW treatment, the temperature was increased by + 5 °C during HW1 and HW2, with a + 2.5 °C gradual increase during the first day of HW1 and HW2. Calculated from the PAR measurements (Fig. 1B), daily light integral (DLI) varied from 4.33 ± 0.09 (d26) to 13.33 ± 3.15 (d16) mol m^−2^ day^−1^ in the control treatment. It remained relatively constant, except for a peak on d16 and a decrease from d24 to d26. In the HW treatment, it was significantly different than in the control only in the last part of the experiment when it increased by 34% from d21 to d33. In the control treatment, the salinity ranged from 37.42 ± 0.05 (d1) to 37.96 ± 0.16 (d25) (Fig. 1C). It was significantly higher in the HW treatment than in the control by an average of 0.31, 0.51, 0.83, and 0.65% in the Post-HW1, HW2, Post-HW2, and from d21 to d33, respectively. The chl-*a* ranged from 0.43 (d33) to 2.01 µg L^−1^ in the control treatment (d4) (Fig. 1D). Its daily average ranged from 0.46 ± 0.12 (d33) to 1.33 ± 0.12 µg L^−1^. It remained constant at approximately 1 µg L^−1^ for most of the experiment, with lower values during the middle of the experiment, before it increased again from d19 to d23. In the HW treatment, it was significantly higher by 37% than in the control in the HW1, reaching 3.09 µg L^−1^ on d5. It was also significantly higher by 18 and 22% than in the control during the HW2 and the Post-HW2, respectively (Supp. Table 2). Over the entire experiment (d1–d33), the chl-*a* was 10% higher in the HW treatment. However, no statistical differences were found between treatments considering the entire experimental period. Dissolved oxygen concentrations ranged from 8.28 ± 0.05 (d26) to 9.66 ± 0.04 gO_2_ m^−3^ (d1) in the control treatment (Fig. 1E). It decreased slightly from d1 to d9, then remained constant until d25, when it decreased again until d26 before slightly increasing until the end of the experiment. It was significantly lower in the HW treatment compared to the control by 3 to 6% during both HWs (1 and 2) and both Post-HWs (1 and 2, Supp. Table 2).

### Dissolved inorganic nutrient concentrations

**Figure 2:**
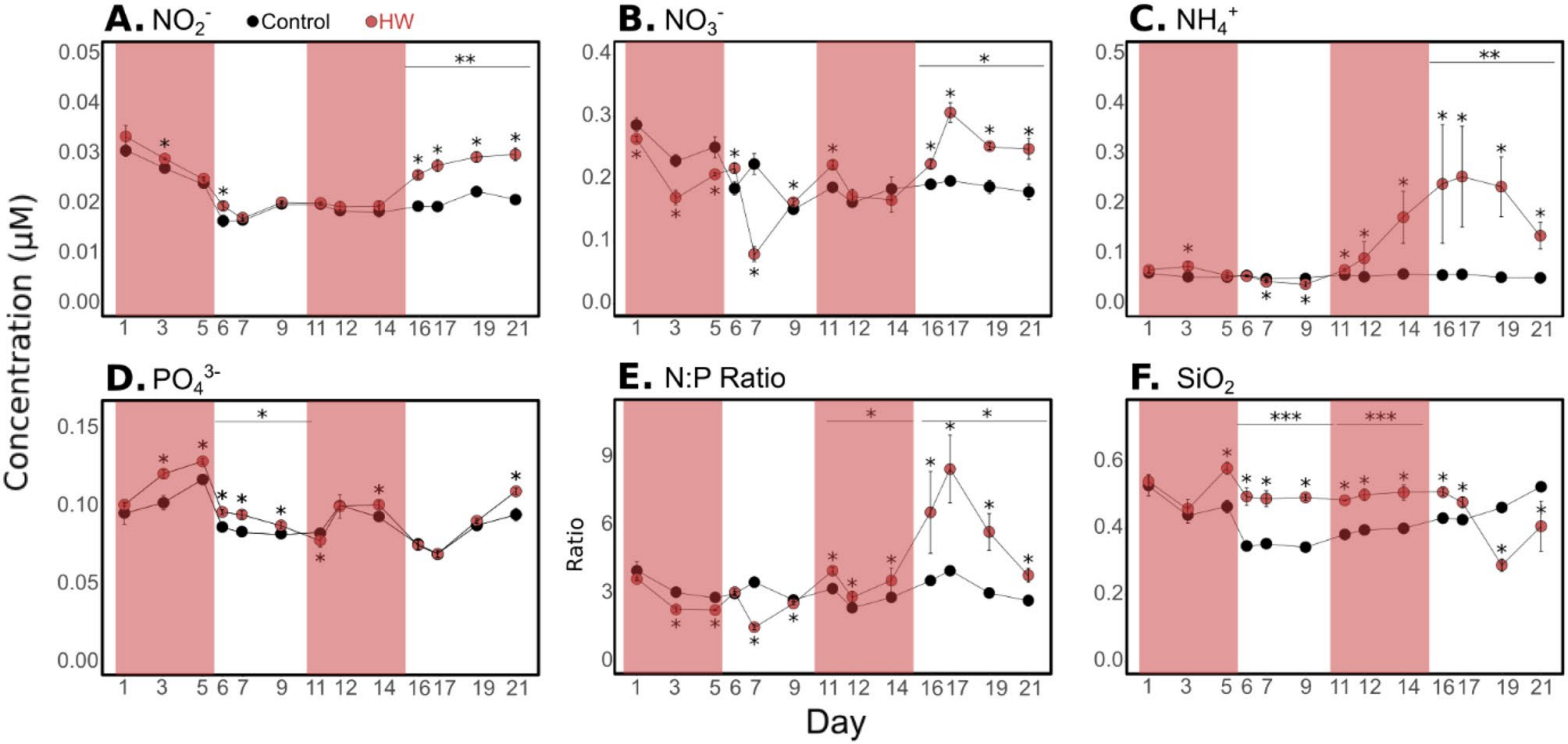
Dissolved inorganic nutrient concentrations. Dissolved nitrite (NO_2_^−^, **A**), nitrate (NO_3_^−^, **B**), ammonium (NH_4_^+^, **C**), orthophosphate (PO_4_^3−^, **D**) concentrations, N:P ratio (**E**) and silicate concentrations (SiO_2_, **F**) until d21 in the control (black) and HW (red) treatments. The red shaded areas represent the HW1 and HW2 periods. The result of a statistical test comparing treatments (*p < 0.05; **p < 0.01) is indicated above each experimental period and the result of a Kruskal–Wallis test comparing treatments day-by-day is indicated for days when a significant difference was found (*p < 0.05). Error bars represent standard deviation apart from d11 to the end for which they represent the range of observations for the HW treatment. Note that dissolved inorganic nutrient concentrations were not measured after d21 due to technical constraints.

In the control treatment, average nitrite (NO_2_^−^) and nitrate (NO_3_^−^) concentrations ranged from 0.016 ± 0.001 to 0.03 ± 0.001 µM and from 0.15 ± 0.01 to 0.29 ± 0.01 µM, respectively (Fig. 2A, B). Silicate (SiO_2_) concentrations were extremely low for the study site, ranging from 0.35 ± 0.01 to 0.53 ± 0.03 µM (Fig. 2F). Ammonium (NH_4_^+^) concentrations were relatively constant, ranging between 0.05 ± 0.003 and 0.06 ± 0.001 µM in the control treatment (Fig. 2C). The orthophosphate (PO_4_^3−^) average concentrations in the control treatment ranged from 0.07 ± 0.01 to 0.12 ± 0.01 µM (Fig. 2D). The NP ratio, calculated as the sum of NO_2_^−^, NO_3_^−^, and NH_4_^+^ concentrations divided by the PO_4_^3−^ concentration, ranged from 2.33 to 3.98 in the control treatment (Fig. 2E).

In the HW treatment, nitrite, phosphate and silicate concentrations were generally significantly higher than that in the control (Fig. 2A, D, F, Supp. Tables 2, 3). Nitrate concentrations were significantly higher during the HW1 and Post-HW1 on specific days (d3 and d6 by 7 and 18%, respectively), and during the Post-HW2 (by 37%). Orthophosphate concentrations were significantly higher during Post-HW1 and on specific days in HW1 and Post-HW2 (d3, d5, d14 and d21 by 8 to 18%). The only exception was found in d11, when concentrations were significantly lower (by 6%). Silicate concentrations were significantly higher in the HW treatment compared to the control at the end of HW1 (25% on d5), during the Post-HW1 (41%), during HW2 (27%) and at the beginning of the Post-HW2 (18% and 12% on d16 and d17, respectively). Meanwhile, the only exceptions were d19 and d21, when concentrations were significantly lower (37% and 23%, respectively). In contrast, nitrate concentrations were significantly lower in the HW treatment than in the control during HW1 (16%) and on d7 (65%). Concentrations were significantly higher on specific days during Post-HW1 and HW2 (d6, d9, d11) and during Post-HW2 (by 8 to 37%, Fig. 2B, Supp. Tables 2, 3). Ammonium concentrations substantially increased in the HW treatment compared to that in the control from d12 until d21, being significantly higher in the HW2 and Post-HW2 (by 94 and 292%, respectively, Fig. 2C, Supp. Tables 2, 3). They were significantly higher than in the control during the HW1 on d3 (40%), whereas in Post-HW1 they were significantly lower on d7 and d9 by 12 and 22%, respectively. Therefore, the NP ratio was significantly lower in the HW treatment than in the control by 6 to 58% on specific days during HW1 and Post HW1 (d3, d5, d7 and d9, Fig. 2E, Supp. Tables 2, 3), as the nitrate concentrations. Driven by the ammonium trend, it significantly increased during the HW2 and Post-HW2, being significantly higher than in the control (by 24 and 86%, respectively).

### Gross primary production, respiration, phytoplankton growth and loss rates

**Figure 3:**
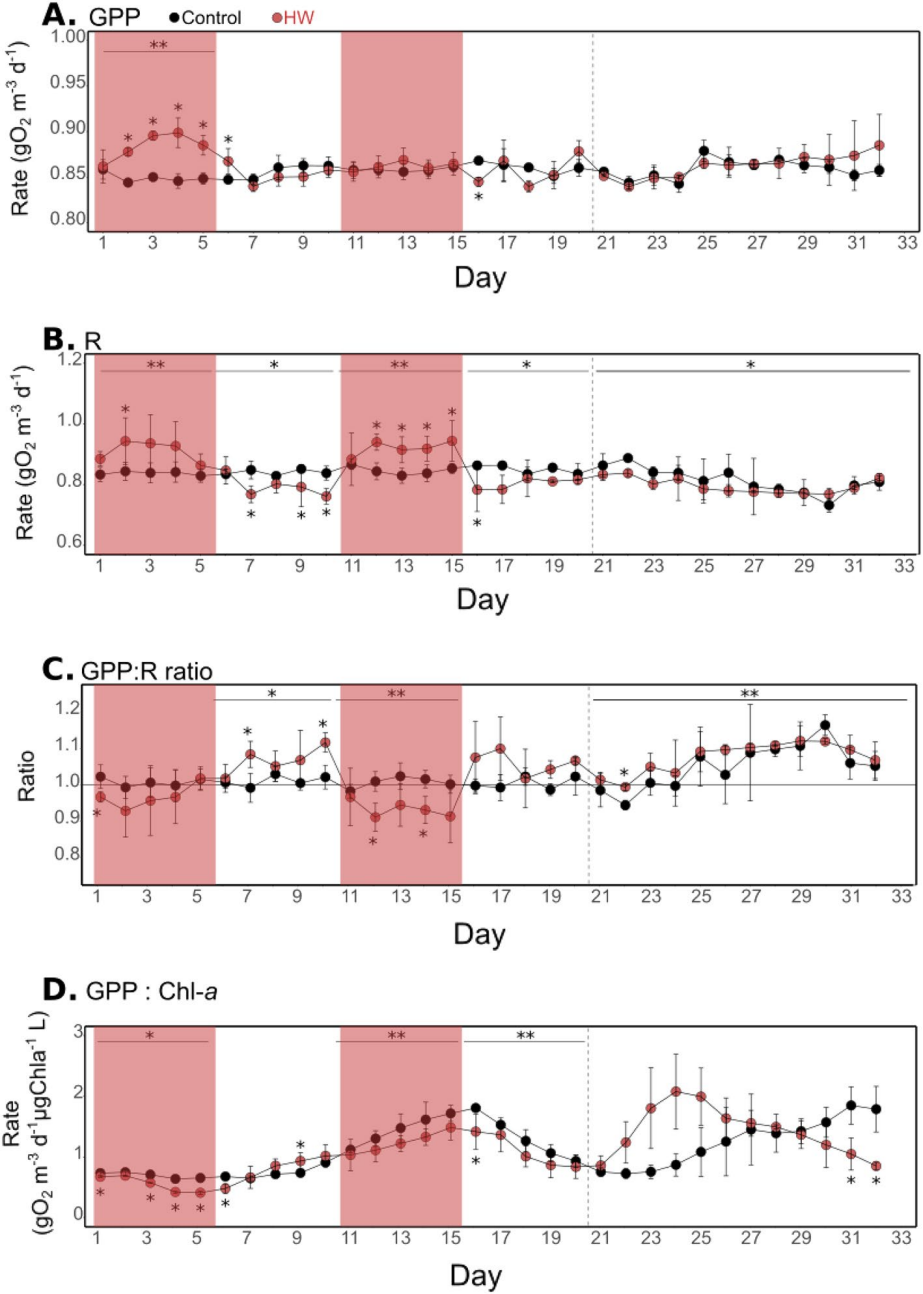
Oxygen metabolism parameters. Gross primary production (GPP, **A**), community respiration (R, **B**), GPP:R ratio (**C**), and GPP normalized by chlorophyll-*a* (**D**) over the course of the experiment in the control (black) and HW (red) treatments. The red shaded areas represent the HW1 and HW2 periods. The result of a statistical test comparing treatments (*p < 0.05; **p < 0.01) is indicated above each experimental period and the result of a Kruskal–Wallis test comparing treatments day-by-day is indicated for days when a significant difference was found (*p < 0.05). Error bars represent standard deviation apart from d11 to the end of the experiment for which they represent the range of observations for the HW treatment. The vertical dashed line indicates the end of the Post-HW2 period (d20).

In the control treatment, GPP and R ranged from 0.84 ± 0.01 to 0.89 ± 0.01 gO_2_ m^−3^ day^−1^ and from 0.94 ± 0.02 (d30) to 1.09 ± 0.01 gO_2_ m^−3^ day^−1^, respectively (Fig. 3A, B). They were both significantly higher in the HW than in the control treatment during HW1 (8 and 7%, respectively). No other significant differences between treatments were found for GPP except on d6 (2%) and on d16 (−3%) (Supp. Tables 2, 3). However, community R was also significantly higher during HW2 (7%), and significantly lower during both Post-HW1 and Post-HW2 (−4% and −5%, respectively) and from d21 to d33 (−2%). Therefore, in the control treatment, the GPP:R ratio ranged from 0.94 ± 0.01 (d22) to 1.17 ± 0.03 (d30), with an average value of 1.02 ± 0.04 (Fig. 3C). The GPP:R ratio was significantly higher in the HW treatment compared to the control during the Post-HW1 (+ 4%) and from d21 to d32 (+ 2%). In contrast it was significantly lower in the HW2 (−6%) (Supp. Table 2). In the HW treatment, it was 1.03 ± 0.07 on average. Finally, in the control treatment, the GPP:chl-*a* ratio ranged from 0.64 ± 0.06 (d4) to 1.85 ± 0.30 gO_2_ m^−3^ day^−1^ µgChl*a*^−1^ L (d31) (Fig. 3D). It was significantly lower in the HW treatment than in the control by 15 to 52% during HW1, HW2, Post-HW2, and on d6, d31 and d32, while being significantly higher only on d9 (+ 26.3%) (Supp. Tables 2, 3).

**Figure 4:**
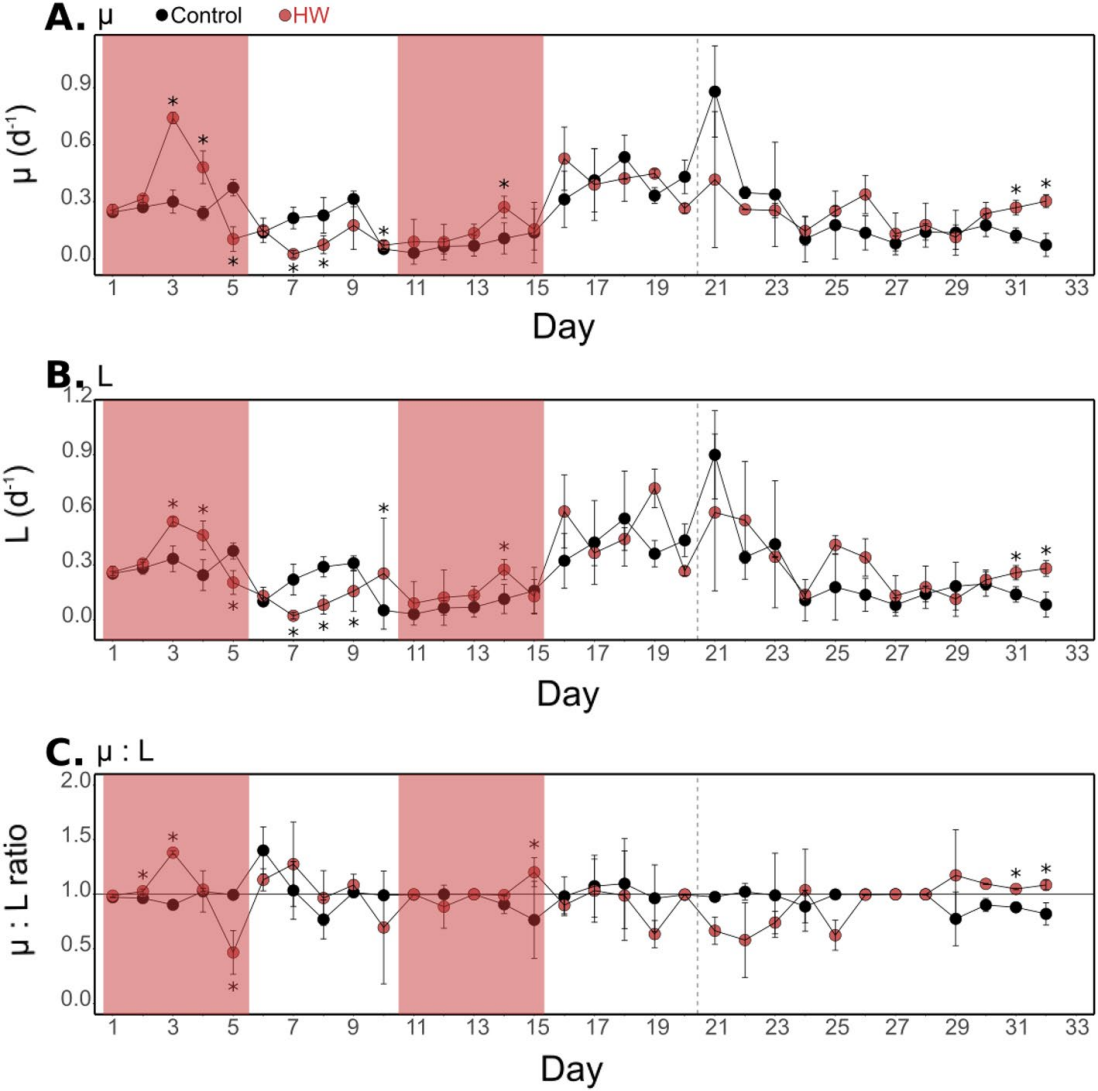
Phytoplankton growth and loss rates. Phytoplankton growth rate (µ, **A**), phytoplankton loss rate (L, **B**), and µ:L ratio (**C**) over the course of the experiment in the control (black) and HW (red) treatments. The red shaded areas represent the HW1 and HW2 periods. The result of a Kruskal–Wallis test comparing treatments day-by-day is indicated for days when a significant difference was found (*p < 0.05). Error bars represent standard deviation apart from d11 to the end for which they represent the range of observations for the HW treatment. The vertical dashed line indicates the end of the Post-HW2 period (d20).

Estimated from the chl-*a* fluorescence data, µ and L displayed similar dynamics. In the control treatment, µ ranged from 0.03 ± 0.01 (d11) to 0.88 ± 0.24 day^−1^ and L ranged from 0.03 ± 0.01 to 0.90 ± 0.24 day^−1^ (Fig. 4A,B). During HW1, µ and L were significantly higher in the HW treatment than in the control on d3 and d4. Then µ and L became significantly lower on d5 and during Post-HW1 (d7 and d8 for µ and also d9 for L). Significantly higher µ and L rates were observed sporadically during Post-HW1 and during HW2 (on d10 and d14 from 39 to 316%), and at the end of the experiment (d31 and d32). Therefore, the mean µ:L ratio ranged from 0.77 ± 0.35 (d15) to 1.40 ± 0.22 (d6) in the control treatment (Fig. 4C). In the HW treatment, it was significantly higher than in the control by 7 to 57% at the beginning of HW1 (on d2 and d3), on d15, and at the end of the experiment (d31 and d32) while being significantly lower only on d5 (53%). The mean µ:L ratio was higher than one almost twice as frequently as that in the control (13 days out of 32 compared with 7 days out of 32 in the control).

### Phytoplankton community structure from pigment and size-fraction analyses

**Figure 5:**
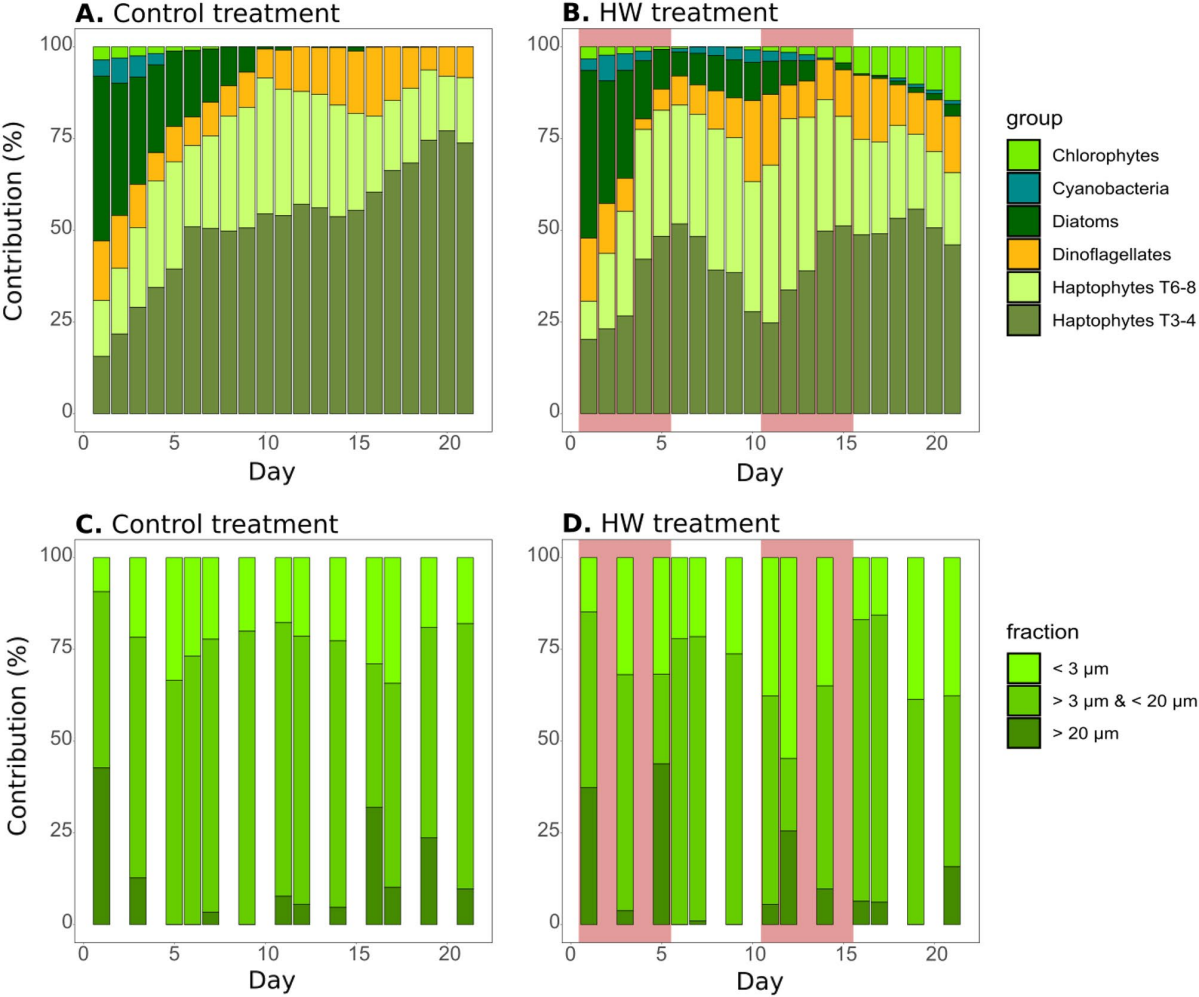
Phytoplankton community and size structure. Relative contribution of phytoplankton groups derived from pigment composition and CHEMTAX analyses in the control (**A**) and HW (**B**) treatments over the course of the experiment, and relative contribution of chlorophyll-*a* size fractions of < 3 µm, between 3 and 20 µm and > 20 µm in the control (**C**) and HW (**D**) treatments over the course of the experiment. The red shaded areas in (**B,D)** represent the HW1 and HW2 periods.

The phytoplankton community structure was assessed using CHEMTAX analysis of taxonomic pigment concentrations and fractioned chl-*a*. In the control treatment, a clear shift in dominance occurred with time, as diatoms dominated the community on d1 (45%), but completely disappeared from d16 until the end of the experiment (Fig. 5A). Similarly, contributions from chlorophytes and cyanobacteria decreased over time, starting at 4% for both groups on d1 and disappearing after d8 for chlorophytes and d5 for cyanobacteria. In contrast, the relative contribution of haptophytes Type 3–4 increased over time, starting at 16% on d1 and reaching 73% on d21. The relative contributions of dinoflagellates and haptophytes Type 6–8 also varied over time, but to a lesser extent, ranging from 6 and 15% (d19) to 19 (d16) and 37% (d10), respectively. In the HW treatment, the average relative contributions of chlorophytes, cyanobacteria and haptophytes Type 6–8 increased compared to the control (by 514, 91, and 21%, respectively). In contrast, haptophytes Type 3–4 contribution decreased in the HW treatment compared to the control (21%) whereas diatoms and dinoflagellates contributed in a similar proportion (Fig. 5B). Specific effects were observed according to the time period and phytoplankton group. The chlorophyte contribution was first significantly lower during HW1 in the HW treatment compared to the control (21%), and its contribution significantly increased during HW2 and Post-HW2 (Supp. Table 2). Significantly higher contributions were also found on specific days during the HW1 for haptophytes Type 3–4 (d4 and d5, both 22%) and haptophytes Type 6–8 (from d3 to d5, from 18 to 31%). However, while haptophytes Type 6–8 also showed significantly higher contributions during Post-HW1, HW2 and Post-HW2 (up to 29%, Supp. Table 2), the contribution from haptophyte Type 3–4 was significantly lower during HW2 and Post-HW2 (up to 29%). Higher contributions were observed in the HW treatment compared to that in the control for cyanobacteria in Post-HW1 and Post-HW2 and for diatoms only in Post-HW2.

For the chlorophyll size fractions, in the control treatment, the fraction 3 µm < chl-*a* < 20 µm was the main fraction contributing between 39 and 95% to the total chl-*a*. Meanwhile, chl-*a* < 3 µm and chl-*a* > 20 µm contributed between a minimum of 9 and 0% to a maximum of 45 and 43%, respectively (Fig. 5C). However, for the 3 µm < chl-*a* < 20 µm, no significant differences were found between treatments, except on d16 when it was significantly higher in the HW treatment than in the control (100%). In contrast, chl-*a* < 3 µm was significantly higher in the HW treatment compared to the control during HW1 (45%), and on d12 and d14 (260 and 79%, respectively). Meanwhile, it was significantly lower only on d7 (4%). Chl-*a* > 20 sharply and significantly increased only on d5 in the HW treatment compared with the control, before returning to the control level and being significantly lower than the control on d16 and d19 (81 and 100%, respectively). Overall, in the HW treatment, chl-*a* < 3 µm contributed between 15 and 93% to total chl-*a* (d1 and d12, respectively), the fraction 3 µm < chl-*a* < 20 µm contributed between 22 and 93% (d12 and d6, respectively), while chl-*a* > 20 µm contributed between 0% (d6, d9, d19) and 46% (d5) to total chl-*a* (Fig. 5D).

### Relationships between the responses of plankton processes with environmental and phytoplankton community parameters

**Figure 6:**
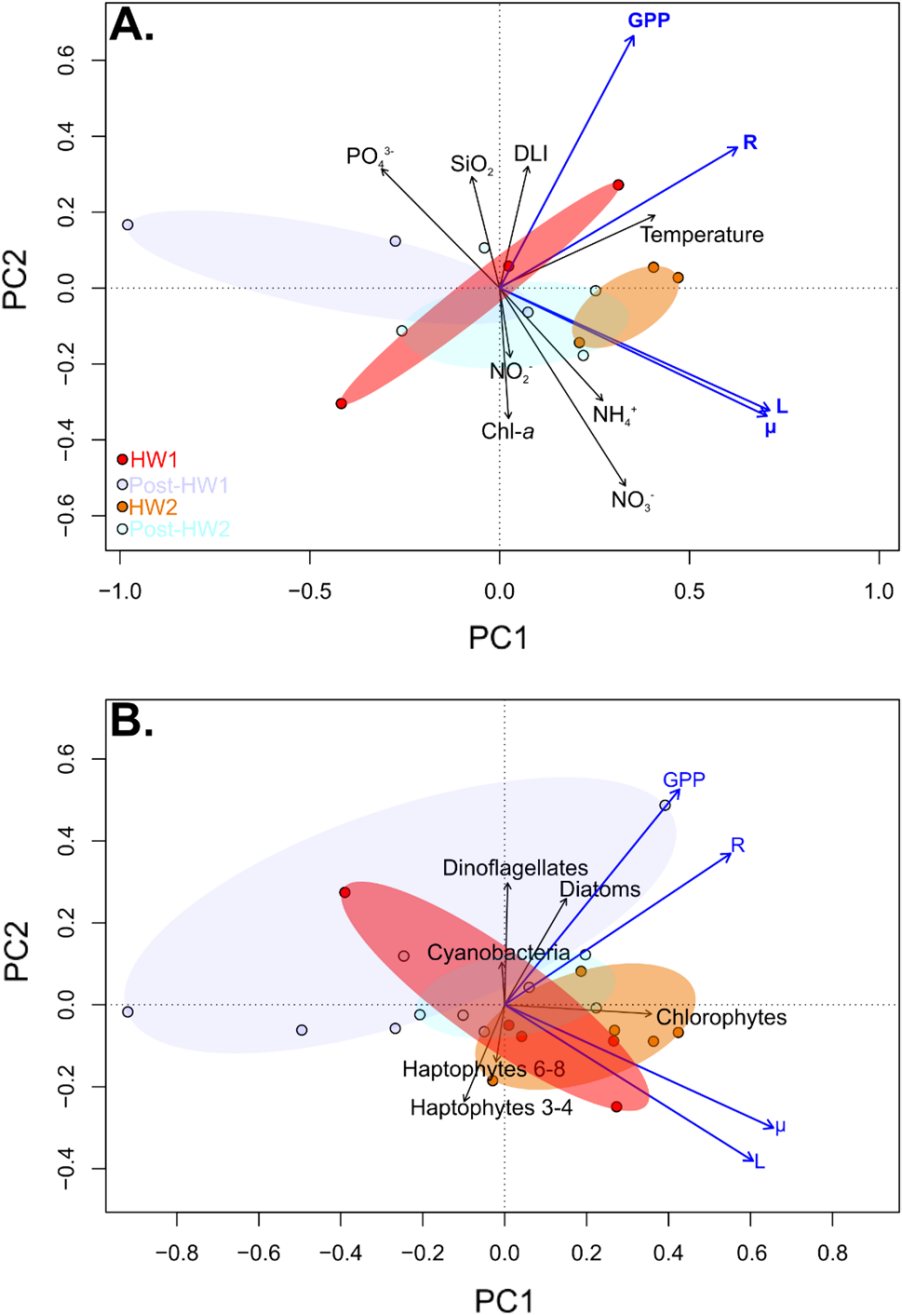
Principal Component Analyses (PCA) biplots of the logarithm response ratio (LRR) of plankton processes with environmental and biological parameters (**A**) and with phytoplankton community structure (**B**) from d1 to d21. Variables are represented with arrows and experimental days as individuals. The *envfit* function from the *vegan* package was used to fit the environmental and biological parameters (**A**) and phytoplankton community structure (**B**) onto the PCA ordination plots. Ellipses are represented in red, orange, dark and light blue for the HW1, HW2, Post-HW1 and Post-HW2 periods, respectively. Plankton processes are indicated in blue, *GPP* gross primary production, *R* respiration, *µ* phytoplankton growth rate, *L* phytoplankton loss rate. Environmental and biological parameters which were included in the analysis were: temperature, *DLI* daily light integral, *chl-a* chlorophyll-*a* concentration, and nutrient concentrations. Phytoplankton community structure parameters which were included in the analysis were CHEMTAX contributions of chlorophytes, cyanobacteria, diatoms, dinoflagellates, haptophytes pigment type 3–4 and haptophytes pigment type 6–8. Note that the number of data included differs from (**A**) to (**B**) as dissolved inorganic nutrients and phytoplankton community structure sampling frequencies were different.

Principal component analyses (PCA) and ordinary least square linear relationships were used to assess potential relationships between the responses, expressed as logarithm response ratio (LRR), of plankton processes (GPP, R, µ and L) with environmental, biological (temperature, DLI, nutrient concentrations, chl-*a* concentration) and phytoplankton community parameters (group contributions from CHEMTAX). In the PCA performed using plankton processes and environmental parameters, the two first axes represented 91.9% of the total variance (Fig. 6A). The results have shown that R response clustered with water temperature. GPP was better-correlated with DLI and SiO_2_. In contrast, µ and L were orthogonal to GPP and R and was clustered with NH_4_^+^ and NO_3_^−^. The ellipsoids and individual plots of the PCA also indicated that responses during all periods were well-separated along both axes. The PCA was also performed using the plankton processes and phytoplankton community structure responses (Fig. 6B). In this analysis, the two first axes explained 89.3% of the total variance. GPP and R was clustered with diatoms. Conversely, µ and L appeared closer to chlorophytes. The analysis also showed a strong temporal pattern of phytoplankton community structure and of its relationship with plankton processes, as HW1 was likely driven by chlorophytes and haptophytes, HW2 by cyanobacteria and Post-HW1 and Post-HW2 by diatoms, cyanobacteria and dinoflagellates.

Ordinary least square linear relationships were also assessed over the entire experiment and significant relationships were found for GPP with R, µ with L, daily chl-*a* with both GPP and R, GPP with dinoflagellates and haptophytes Type 3–4, and µ with NO_3_^-^ and NP ratio (Table 1). During HW1, µ was linearly related to L and chl-*a* < 3 µm, and GPP to haptophytes Type 6–8 (Table 1). During HW2, only µ and L were linearly related (Table 1). Meanwhile, during Post-HW1, significant linear relationships were found for µ with L, and GPP with chl-*a* and with NO_2_^−^ concentration (Table 1). During Post-HW2, the only significant linear relationships were µ with L and with haptophytes Type 3–4 (Table 1).

**Table 1:** Ordinary least squares linear relationships between logarithm response ratio (LRR) of plankton processes (GPP, R, µ and L) with environmental and biological (temperature, DLI, nutrient concentrations and chl-*a* concentration) and phytoplankton community parameters (group contributions from CHEMTAX) over the entire experiment (d1–d32 for plankton processes and temperature, DLI and chl-*a*, d1–d21 for nutrient concentrations and phytoplankton structure parameters), over the HW1, post-HW1, HW2 and post-HW2 periods.

	Entire experiment	HW1	Post-HW1	HW2	Post-HW2
GPP ~ R	y = 0.2x + 0.01***	n.s	n.s	n.s	n.s
µ ~ L	y = 0.87x − 0.02***	y = 1.71x − 0.05***	y = 0.65x − 0.17*	y = 0.72x + 0.07*	y = 0.73x − 0.02*
GPP ~ chl-*a*	y = 0.06x − 0.01**	n.s	y = 0.07x − 0.01*	n.s	n.s
R ~ chl-*a*	y = 0.17x − 0.01*	n.s	n.s	n.s	n.s
GPP ~ NO_2_^-^	n.s	n.s	y = 0.24x − 0.01*	n.s	n.s
µ ~ NO_3_^−^	y = 1.28x − 0.02*	n.s	n.s	n.s	n.s
µ ~ NP ratio	y = 1.16x − 0.08*	n.s	n.s	n.s	n.s
µ ~ chl-*a* < 3 µm	n.s	y = 13.8x − 2.34*	n.s	n.s	n.s
L ~ chl-*a* < 3 µm	n.s	y = 6.85x − 1.16*	n.s	n.s	n.s
GPP ~ Dino	y = −0.02x + 0.004*	n.s	n.s	n.s	n.s
GPP ~ Hapto68	n.s	y = 0.08x + 0.014*	n.s	n.s	n.s
GPP ~ Hapto34	y = 0.04x + 0.007*	n.s	n.s	n.s	n.s
µ ~ Hapto34	n.s	n.s	n.s	n.s	y = 4x + 0.52*

Only significant relationships (p < 0.05) in at least one tested period are mentioned in the table. Significance level are mentioned with the * symbol (*p < 0.05, **p < 0.01, ***p < 0.001). *n.s*. not significant.

*GPP* gross primary production, *R* respiration, *µ* growth rate, *L* loss rate, *chl-a* chlorophyll-*a*, *Dino* dinoflagellates, *Hapto68* haptophytes type 6–8, *hapto34* haptophytes type 3–4.

## Discussion

The aim of this study was to investigate the functional responses of coastal Mediterranean plankton communities to consecutive experimental HWs, with a focus on oxygen metabolism, phytoplankton growth, loss and community structure. Strong significant effects on GPP, R, µ and L were found during the first HW, whereas the effects of the second HW on these processes were substantially reduced compared to those of the first HW, apart from community R, which responded similarly to both HWs. An important restructuring of phytoplankton succession also occurred as a consequence of both HWs.

The first HW significantly increased phytoplankton biomass, its µ and L, as well as GPP and community R by 7 to 33%. These positive effects of HW1 are congruent with the theoretical positive effect of warming on metabolic rates^40^, with observations reporting higher phytoplankton biomass, metabolism and community R at higher temperatures in various regions and systems^10,11,41^, and with other experiments showing similar positive effects of experimental warming^32,33,42–44^. In the present study, it is likely that the increase in phytoplankton biomass resulted in the significant decrease in NO_3_^-^ concentration during HW1 in the HW treatment, indicating a more rapid depletion of N than P under elevated temperature. This suggests that N rather than P is a controlling factor for plankton communities, which is confirmed by the very low NP ratios reported in the experiment and is congruent to a typical post-bloom situation in Thau lagoon during late spring^38,45^. In line with this, the phytoplankton growth response was found to be linearly related to both the NO_3_^-^ and NP ratio responses over the course of the experiment. Furthermore, the dissolved phosphorus pool could have been replenished through breakdown of cells and excretion by zooplankton^46^ given that the phytoplankton loss rate was found to be enhanced by on average 19% during HW1, thereby potentially preventing a potential P limitation. Nevertheless, it should be noted that growth was calculated from daily cycles of chl-*a* with the assumption that daily variations of chl-*a* are only due to growth and losses from mortality and sedimentation. However, other factors may have affected chl-*a* cycles, such as potential changes in cellular physiology related to variations in inorganic nutrients and light availabilities^47^, thus it cannot be excluded that estimated growth rates were under- or overestimated depending on some environmental conditions in the mesocosms.

The second HW had a less pronounced effect on phytoplankton biomass and related processes compared to the first one, as the positive effect on daily chl-*a* during HW2 was half as much as that of HW1, and no significant effect was found for GPP, µ nor L. Given that daily light and most nutrient concentrations were within the range of those observed during HW1, the difference in phytoplankton responses between HWs seems unrelated to bottom-up controls. Instead, this could be due to changes in the community composition toward better thermally adapted taxa, as phytoplankton had already experienced high temperature during HW1 a few days before.

Given that GPP and R were enhanced to similar extents (+ 8 and + 7% respectively) during HW1, the GPP:R ratio was not significantly different between treatments. This indicates that the first HW did not alter the metabolic balance of the planktonic community of Thau lagoon. This result is in contrast to what was already observed under a moderate HW (+ 3 °C) in late spring/early summer in Thau lagoon which shifted the system toward heterotrophy^32^. This is likely because of different conditions at the start of the experiments. While the stronger warming effect on R than on GPP reported by Soulié et al.^32^ is congruent with theoretical predictions, similar magnitudes of the positive effects on GPP and R found in the present study during HW1 suggest a strong dependence of community R on primary production. However, these similarities in the effect magnitude of HW1 on GPP and R may only be related to temperature, independent from a relationship between primary production and respiration.

Indeed, during HW2, only community R responded positively as it was enhanced by 7%, which is similar to its response during HW1, whereas GPP did not change. This result confirms the well-known stronger role of temperature in driving respiration compared to that on photosynthesis, in line with its higher activation energy^40,48^. The concomitant accumulation of NH_4_^+^ in the HW mesocosms starting during HW2 supports the hypothesis of a positive response of heterotrophic bacteria, as NH_4_^+^ is one of the main products of organic matter remineralization by bacteria^49^. This may have resulted in the positive response of R, given that bacteria often strongly contribute to the whole community R^50^. The important increase of NH_4_^+^ could also be related to higher zooplankton excretion in the HW treatment compared to the control^51^. This is congruent with the higher phytoplankton loss rate found on d14. In this regard, information concerning other communities, such as zooplankton and bacteria, could be helpful in understanding the nutrient dynamics that was reported during the experiment. Overall, given the increase of R and the absence of response of GPP, HW2 shifted the metabolic balance (GPP:R) of the system toward heterotrophy. Therefore, the findings of the present study suggest that consecutive HWs could result in short-term (a timescale of a few days) deoxygenation of the water column. This could contribute to the strengthening of hypoxic and/or anoxic events that have already occurred in Mediterranean coastal lagoons^52,53^.

Important changes were also reported during Post-HW1 and Post-HW2. Chl-*a*, phytoplankton growth rate and GPP strongly decreased and returned to the control level within one day after the end of HW1. This suggests a stronger control through nutrient bottom-up regulation of phytoplankton during Post-HW1 than during HW1, given that both phytoplankton growth rate and NO_3_^-^ concentrations were lower in the HW treatment than in the control during Post-HW1. Comforting the hypothesis of bottom-up rather than top-down control on phytoplankton biomass at this time, the phytoplankton loss rate was significantly lower than in the control, refuting the hypothesis of stronger zooplankton grazing pressure.

Indeed, phytoplankton loss rate (which includes grazing, viral lysis, sedimentation and natural death^12,13^) was significantly lower in the HW treatment than in the control at the end of HW1 and during Post-HW1. This result is congruent with those obtained during an experiment in the Baltic Sea in which warm and low-nutrient conditions favored copepod grazing on ciliates, which reduced the grazing pressure on phytoplankton through a trophic cascade mechanism^54^. This is also in line with the findings of Courboulès et al.^30^ who found that experimental warming lowered grazing on phytoplankton in Thau lagoon fall community, potentially because of lower nutritional quality of phytoplankton under warming due to low nitrate availability. Nevertheless, our study suggests a low resilience of phytoplankton loss factors, such as predators and viruses, for a few days after an intense short-lived HW in Thau lagoon.

In the present study, consecutive HWs altered the phytoplankton community structure. HW1 favored haptophytes Type 6–8 and Type 3–4 at the expense of chlorophytes, and modified phytoplankton size structure with increases in the < 3 and > 20 µm fractions. Haptophytes Type 6–8 consist of 4’-keto-19’-haxanoyloxyfucoxanthing containing species, such as *Emiliania* *huxleyi*, while haptophytes Type 3–4 consist of species containing an important amount of fucoxanthin, such as *Isochrysis* *galbana* and *Prymnesium* *parvum*^55^. Pigment biomarkers of haptophytes were already shown to be promoted during an experimental HW in Thau lagoon and to make an important contribution to GPP^32,33^. Their advantage under warming could be from their competitiveness over other groups in certain nutrient and temperature conditions^56^. Given that haptophytes are important components of phytoplankton worldwide, being potentially toxics and/or mixotrophic^57,58^ and forming blooms that contribute substantially to regional biogeochemical cycles^59,60^, such positive impact of HWs on their relative abundance could significantly alter the functioning of Mediterranean coastal ecosystems in the future. In Thau lagoon, it was already reported that haptophytes could play a dominant role in the phytoplankton community, especially following early spring diatom blooms^61^. Our results suggest that future HWs occurring after spring blooms could exacerbate this role within Thau lagoon phytoplankton community. Nonetheless, changes in phytoplankton community composition reported in the present study need to be interpreted with caution, as they were assessed with pigment concentrations, which cellular content is known to be sensitive to changes in light and nutrient conditions^62^.

During Post-HW1, an important increase in cyanobacteria relative abundance was observed in the HW treatment. Cyanobacteria were most likely favored because of their competitiveness under low nutrient conditions^63,64^. However, they could also have taken advantage of a switch in grazing pressure toward larger cells, considering that phytoplankton seemed dominated by larger cells, such as diatoms and haptophytes. Our results are also in line with those of Collos et al.^65^, Bec et al.^66^ who found that the emergence of cyanobacteria in Thau lagoon is related to low nutrient conditions and temperature increases. Cyanobacteria were shown to be of great importance in the functioning of the food web of Thau lagoon^67^, and the findings of the present study indicates that this role could be amplified in the future in response to consecutive HWs.

During Post-HW2, a strong increase in the relative abundance of chlorophytes, which contributed up to 14% of the total phytoplankton community, was reported in the HW treatment. Meanwhile, the haptophytes Type 3–4, which were largely dominating the community in the control treatment, were significantly depressed. Green algae such as chlorophytes have already been shown to be promoted by higher winter temperature in Thau lagoon^68^. They are known to potentially form abnormal massive harmful blooms with profound ecological and economic consequences in the lagoon^69,70^. This suggests that consecutive HWs could result in dysfunctional situations, which could be detrimental for higher trophic levels and for the entire lagoon ecosystem functioning. Indeed, this increase of chlorophyte relative contribution is similar to the shift in Thau lagoon phytoplankton community toward picophytoplankton that was reported during a natural HW in 2019, which had profound consequences for oyster populations^71^. In the present study, while a diatom-to-haptophyte succession was observed in the control treatment over time, which is typical of post-spring bloom conditions in the lagoon^61^, the increase in chlorophyte relative abundance after HW2 indicates that consecutive HWs could substantially change the normal phytoplankton community succession, emphasizing on the structural consequences of such extreme events.

In conclusion, in the present study, the use of high-frequency sensors immersed in in situ mesocosms allowed the detection of complex responses of key plankton processes toward consecutive HWs on the spring communities of a Mediterranean coastal lagoon. Our study has highlighted a significant effect of the first HW on most plankton processes, enhancing both primary production and respiration. This effect was stronger compared to the effect of the second HW on phytoplankton-related processes, suggesting a thermal acclimation and complex interactions among plankton communities and with the environment, which may indicate that prior exposure to a HW might mitigate the consequences of subsequent exposures. Our study also found different responses over short (few days) to medium (few weeks) timescales, notably concerning respiration, and so metabolic balance of the system. This has confirmed the known role of temperature in structuring spring plankton communities in Thau lagoon^68^. Therefore, the present study indicates that consecutive HWs could have impacts of various magnitude on coastal ecosystem functioning because of the different response times of phytoplankton and of its controlling factors. These are not usually considered in global predictions of increased HW frequency consequences on plankton communities.

Even if mesocosm experiments cannot fully replicate natural environments and their complexity^72,73^, they represent a mandatory step in understanding how climate change-related drivers affect plankton community structure and functions. This then allows the development of further predictions regarding the evolution of ecosystems under such climate change. Although the consequences of climate change have predominantly been studied through the lens of constant perturbations, an increasing number of studies have focused on understanding how episodic climate change-related disturbances affect ecosystems^74,75^. The results of the present study contribute to a broader understanding of the effects of consecutive HWs on plankton assemblages and will prove useful in refining model predictions regarding the evolution of coastal ecosystems under future climate change.

## Methods

### Study site and in situ mesocosm experiment setup

The in situ mesocosm experiment was performed in spring of 2022 in Thau lagoon, a Mediterranean coastal lagoon with a mean depth of 4 m^38^. The mesocosms were installed into the lagoon at the Mediterranean Platform for Marine Ecosystems Experimental Research facilities (MEDIMEER, 43°24′53′′ N 3°41′16′′ E). The experiment lasted 33 days, from April 28th to May 31st, 2022. The mesocosms were 230 cm high and 120 cm wide bags and were made of nylon-reinforced transparent vinyl acetate polyethylene (Insinööritoimisto Haikonen Ky, Finland). To avoid external input, each mesocosm was covered with a transparent polyvinyl-chloride dome that transmitted 73% of the received photosynthetically available radiations (PAR). On April 28th (d0), mesocosms were filled simultaneously with 1700 L of lagoon water that was gently pumped at 1 m depth, screened through a 1000-µm mesh to remove large particles and organisms, before being pooled in a large container and distributed simultaneously to all the mesocosms through six parallel pipes. As mesocosms were placed directly in the lagoon and subjected to natural water movements, the water column in the mesocosms was naturally mixed with winds and currents of the lagoon.

Two treatments, differing in their water temperature, were applied in triplicate to the six mesocosms. Three mesocosms served as controls, and had natural lagoon water temperature throughout the experiment. In the three others, referred to as the HW treatment, two consecutive HW were simulated by raising the water temperature at + 5 °C for 5 days compared to the control mesocosms. For each HW, the water was heated at + 2.5 °C for the first day to avoid raising the temperature too sharply, and then at + 5 °C from the second day to the 5th day of each HW. Then, heating was stopped and the water temperature of the HW mesocosms returned naturally to that of the control mesocosms for the next 5 days. Therefore, the first HW, hereafter called “HW1 period”, was applied from d1 to d5. It was then followed by a post-heat wave period without heating (from d6 to d10), hereafter referred to as the “Post-HW1 period”. The second heatwave was applied from d11 to d15 and hereafter referred to as the “HW2 period”. Then, until the end of the experiment, heating was stopped and the temperature of all the mesocosms followed the temperature of the lagoon. To compare with Post-HW1, a 5-day period after the HW2 (d16–d20) was considered and was referred to as the “Post-HW2 period”. The experiment was run for 12 additional days after the end of Post-HW2. To achieve a + 5 °C increase during both HW1 and HW2, a submersible heating element (Galvatek) was immersed at 1 m depth in each HW mesocosm, and was automatically controlled to adjust constantly at + 5 °C compared to the control mesocosms. The detailed heating procedure can be found in the studies of Nouguier et al.^76^ and Vidussi et al.^28^. Due to a technical problem, heating reached + 8 °C for several hours instead of the targeted + 2.5 °C on d11 in one of the HW mesocosms. Therefore, the data from this replicate was removed from the analysis from d11 to the end of the experiment. Note that manual sampling of the mesocosms was stopped after d21 but the experiment was kept running only by the use of sensors measuring for 12 more days to assess a longer recovery trend.

### High-frequency sensor data acquisition, calibration and correction

In each mesocosm, a set of high-frequency sensors was immersed to a depth of 1 m. Each set included a dissolved oxygen sensor (Aanderaa 3835), chlorophyll fluorometer (WetLabs ECO-FLNTU), conductivity sensor (Aanderaa 4319), and spherical underwater quantum sensor (Li-Cor Li-193). Three temperature probes (Campbell Scientific Thermistore Probe 107) were placed in each mesocosm at three different depths (0.5, 1 and 1.5 m). Measurements were recorded every minute during the entire experiment.

The dissolved oxygen, chl-*a* fluorescence, conductivity and water temperature sensors were calibrated prior to the experiment. Chl-*a* fluorescence and oxygen data were also corrected using discrete measurements performed during the experiment^32,77,78^. The detailed procedures and a comparison between chl-*a* fluorescence sensor and discrete measurements with high performance liquid chromatography (HPLC) are presented in the Supplementary material.

### Daily light integral (DLI) from high-frequency PAR measurements

High-frequency PAR measurements performed at 1 m depth in the mesocosms were used to calculate the daily light integral (DLI), which corresponds to the daily amount of photosynthetically active photons received on a 1 m^2^ surface over a 1 day period^79^, with Eq. (1), where the DLI is expressed in mol m^−2^ day^−1^, the mean PAR between sunrise and sunset in µmol m^−2^ s^−1^, and the day length in h.

1 $$DLI= \frac{mean PAR \times day length \times 3600}{1 \times 1{0}^{6}}$$

### Phytoplankton growth (µ) and loss (L) rates from high-frequency chlorophyll-*a* fluorescence measurements

Corrected and calibrated high-frequency chl-*a* fluorescence measurements were used to estimate µ and L, using a method detailed in Soulié et al.^33^. Each chl-*a* fluorescence cycle was separated into periods during which chl-*a* fluorescence increased and periods during which it decreased. An exponential fit was applied to the chl-*a* fluorescence data for each period. Then, µ and L were estimated, under the assumptions that changes in chl-*a* are only due to phytoplankton losses during the night and to both growth and losses during the day^80^. The detailed calculations and a comparison between phytoplankton growth rates obtained from sensor data and daily net changes obtained from discrete measurements with HPLC are presented in the Supplementary material.

### Gross primary production (GPP) and community respiration (R) from high-frequency dissolved oxygen measurements

Corrected and calibrated high-frequency dissolved oxygen concentration measurements were used to estimate GPP and R, using the method described in Soulié et al.^78^. Derived from the free-water diel oxygen technique^81^, this method was specifically developed for mesocosm experiments and to consider the variability in respiration rates between day and night. Each dissolved oxygen cycle was separated into periods during which the dissolved oxygen concentration increased and periods during which it decreased. Then, the dissolved oxygen data from each period were smoothed using a 5-point sigmoidal model. These smoothed data were then used to estimate the physical exchange of oxygen between water and the atmosphere, as well as GPP and R. The detailed calculations are presented in the Supplementary material.

### Manual sampling of the mesocosms for dissolved inorganic nutrient concentrations, and phytoplankton community structure

Each mesocosm was manually sampled in the morning (09:00) using a Niskin water sampler immersed at depth of 1 m. Before each sampling, the water column of the mesocosms was gently mixed. Sub-samples were then taken daily from the Niskin water sampler for phytoplankton pigment composition from d1 to d21, and every two to three days from d1 to d21 for dissolved inorganic nutrient concentrations, and phytoplankton size fractions.

To measure dissolved inorganic nutrient concentrations, sub-samples (50 mL) from the Niskin water sampler were put into acid-washed polycarbonate bottles and filtered with a 0.45 µm filters (Gelman). The samples were then stored in a polyethylene tube at –20 °C until further analysis. Analyses for nitrate, nitrite, orthophosphate and silicate were performed using an automated colorimeter (Skalar Analytical) and following the protocol detailed by Aminot and Kérouel^82^. Quantification limits were 0.125 µM for nitrate, 0.008 µM for nitrite, 0.021 µM for orthophosphate, and 0.123 µM for silicate. Analyses for ammonium were performed using a spectrofluorometer (Perkin Elmer LS45) and following the fluorometric method described by Holmes et al.^83^.

The phytoplankton community structure was assessed based on the size fractions of chl-*a*^84^ and taxonomic pigment composition. Total chl-*a* sub-samples (300 mL) were collected from the Niskin water sampler in covered high-density polypropylene bottles before being filtered at low ambient light with a low vacuum pump on a glass-fiber filter (Whatman GF/F, 0.7 µm pore size). Two other fractions were filtered through 20 (Merck Millipore Ltd. Nylon Net, 20 µm pore size) and 3 µm (Whatman Nuclepore, 3 µm pore size), respectively. Chl-*a* was then extracted in 90% acetone for at least 12 h at 4 °C^85^. After extraction, measurements were performed using a spectrofluorometer (Perkins Elmer FL6500) calibrated with chl-*a* standards (Sigma-Aldrich C-5753) with ten calibration points ranging from 0 to 183.03 µg L^−1^.

Sub-samples (700–1500 mL) were taken in covered high-density polypropylene bottles for analysis of phytoplankton taxonomic pigment composition. They were filtered on a glass-fiber filter (Whatman GF/F, 0.7 µm pore size) at low ambient light with a low-vacuum pump before being flash-frozen in liquid nitrogen and stored at −80 °C until analysis. Pigments were then extracted in 2 mL of 95% methanol during 1 h at −20 °C, filters were then sonicated and stored during 1 h at 4 °C^28^. The extracts were clarified by filtering on a glass-fiber filter (Whatman GF/F, 0.7 µm pore size) and directly analyzed by HPLC (Shimadzu), following the method of Zapata et al.^86^. The CHEMTAX 1.95 program^87^ was used to assess the relative contribution of taxonomic groups. An initial biomarker pigment to chl-*a* ratio matrix was created based on previous studies in the Mediterranean^88–91^ (Supplementary material Table 1). To ensure that the optimized pigment ratios were used, 60 randomized copies of the initial ratio matrix were created and used as multiple starts for the iterative process^92^. These randomized matrices were generated by applying a random factor *F*, calculated using Eq. (2):

2 $$F=1+0.7\times (R-0.5)$$

with *R* being a random number between 0 and 1 generated using the RAND function in Microsoft Excel^92^. Once each randomized matrix has been used as a starting point for CHEMTAX, the six best results, that is, the six results with the smallest residuals, were used to calculate the average abundance estimates. CHEMTAX parameterizations were performed as follows: iteration limit was set as 200, epsilon limit as 0.0001, initial step size as 10, set ratio as 1.3, cutoff step as 1000, elements varied as 5, subiterations as 1, weighting as 3 and weight bound as 30. Based on pigments identified in the samples, six different phytoplankton groups were discriminated: chlorophytes, cyanobacteria, diatoms, dinoflagellates pigment Type I (containing peridinin), haptophytes pigment Type 6–8 (containing 19’-hexanoyloxyfucoxanthin, 19’-butanoyloxyfucoxanthin and 4-keto-19’-heaxanoyloxyfucoxanthin) and haptophytes pigment Type 3–4 (containing fucoxanthin)^55,86^.

### Statistical analyses

To test the differences between the control and HW treatments, one-way repeated-measures analysis of variance (RM-ANOVA) was performed over the entire experiment (d1-d33) and over specific periods (HW1, HW2, Post-HW1, Post-HW2) with treatment as a fixed factor and time as a random factor, and considering an autoregressive process of order 1^72,93^. The confidence level was set as 0.05, meaning that a *P*-value less than or equal to 0.05 was considered representative of a significant treatment effect. Assumptions of normality of residuals and homoscedasticity were checked using the Shapiro–Wilk and Levene tests, respectively. When these assumptions could not be met after data transformation (logarithmic, exponential or square-root), a non-parametric Kruskal–Wallis test was performed on the ranks instead. Kruskal–Wallis tests were also performed individually for each experimental day to assess the treatment effects on specific days. Multivariate (Principal Component Analyses (PCA)) and univariate (ordinary least squares linear relationships) analyses were used to evaluate potential relationships between the responses to the HW of plankton processes (GPP, R, µ and L) with environmental and biological (temperature, DLI, nutrient concentrations, chl-*a* concentration) and phytoplankton community parameters (group contributions from CHEMTAX). These were performed using the effect of the treatment, expressed as the Logarithmic Response Ratio (LRR) calculated using Eq. (3):

3 $$LR{R}_{X}=\mathrm{log}(\frac{{X}_{HW}}{{X}_{C}})$$

with $LR{R}_{X}$ the LRR of variable X, X_HW_ and X_C_ the values of variable X in the HW and control treatments, respectively. The *envfit* function from the *vegan* package was used to fit the environmental, biological (temperature, DLI, nutrient concentrations, chl-*a* concentration) and phytoplankton community composition (group contributions from CHEMTAX) onto the PCA ordination plots. All the data management and analyses were performed using R (version 4.0.1) and CHEMTAX (version 1.9.0.5) software.

## Supplementary Information

Supplementary Information.

## Data Availability

The datasets used and/or analysed during the current study are available from the corresponding author on reasonable request.
