# Supplementary material for: Functional and structural responses of plankton communities toward consecutive experimental heatwaves in Mediterranean coastal waters
Source: Sci Rep. 2023 May 17;13:8050. doi: 10.1038/s41598-023-35311-4 (PMC10192389; doi:10.1038/s41598-023-35311-4)
Supplement: Supplementary file 1 — Supplementary Information. [file 41598_2023_35311_MOESM1_ESM.pdf]

Functional and structural responses of plankton communities toward consecutive experimental heatwaves in Mediterranean coastal waters

Tanguy Soulié[1], Francesca Vidussi[1], Sébastien Mas[2], Behzad Mostajir[1]

[1]MARBEC (MARine Biodiversity, Exploitation and Conservation), Univ Montpellier, CNRS, Ifremer, IRD, Montpellier, France

[2]MEDIMEER (MEDIterranean platform for Marine Ecosystems Experimental Research), OSU OREME, CNRS, Univ Montpellier, IRD, INRAE, Sète, France

Supplementary Material

High-frequency sensor calibration and data correction

The dissolved oxygen sensors were calibrated at three different saturation points (0, 50 and 100%). The 100% saturation point was reached by gently bubbling air into distilled water. The 0 and 50% saturation points were reached by adding potassium metabisulfite into distilled water. The calibrated dissolved oxygen data were then corrected with the salinity and water temperature data according to the procedure detailed in Bittig *et al*. (2018). Finally, data were corrected with discrete oxygen concentration measurements performed with the Winkler titration technique, as detailed in Soulié *et al*. (2021). The fluorescence sensors were calibrated with a monoculture of Dunaliella tertiolecta and at three concentration points, ranging from 0 to 6.78 µg L-1. The reference chl-a concentration was measured using spectrophotometry (FL6500, Perkins Elmer, United States) and the protocol of Ritchie (2006). In addition, high-frequency chl-a fluorescence data were corrected for non-photochemical quenching by linearly interpolating the data from sunrise to sunset (Soulié *et al.* 2022a). Finally, data were also corrected with discrete chl-a concentration measurements performed with high performance liquid chromatography (HPLC, Shimadzu, Japan). Salinity sensors were calibrated at three different salinity points (0, 30 and 40) obtained by adding sodium chloride in distilled water. Salinity data were then smoothed with a 9-point moving average. Water temperature probes were calibrated in a temperature-controlled water bath with six points, ranging from 5°C to 30°C.

Phytoplankton growth (µ) and loss (L) rates from high-frequency chlorophyll-*a* fluorescence measurements

Phytoplankton µ and L were estimated using the corrected chl-*a* fluorescence data and a method detailed in Soulié *et al*. (2022) and following similar principles as in Neveux *et al*. (2003). The daily chl-*a* cycle was separated into two parts: the "increasing period," which starts from sunrise until the fluorescence maximum is reached (generally a few hours after sunset), and the "decreasing period," from the time of maximum fluorescence until the following sunrise. The maximum chl-*a* always occurred several minutes to a few hours after sunset. For each period, an exponential regression was performed using the chl-*a* as y and time as x, following **Eq. S1**:

$$Chla = a \times e^{bt}$$

With $Chla$ the chl-*a* (µg·L^{-1}), a (µg·L^{-1}) and b (min^{-1}) constants, and t the time (min). Following Neveux *et al*. (2003), as there is no growth during the night and mesocosms are enclosed systems, the changes in $Chla$ are assumed to be due to L at night during the "decreasing period." Therefore, during the "decreasing periods," $b = L$, with L in min^{-1}, and during the "increasing periods," $b = \mu - L$. Subsequently, µ (min^{-1}) was calculated using **Eq. S2**:

$$\mu = (\mu - m) + L$$

Finally, L and µ were converted to hr^{-1} by multiplying by 60 to obtain the hourly rates. Then, hourly L rates were multiplied by 24 to obtain daily rates (d^{-1}). Here, we assumed that losses occurred during the entire 24-hr period, and hourly µ rates were multiplied by the duration of the increasing period, in hours, as growth only occurred during the increasing period.

Comparisons between sensor and HPLC measurements

High-frequency chl-*a* measurements obtained with the sensors were compared with daily discrete measurements of chl-*a* concentration obtained with HPLC. To do so, sensor chl-*a* data were averaged for the period 8:30 – 9:30 as HPLC samples were taken daily around 9:00. An ordinary least square linear relationship was assessed between sensor and HPLC data (**Supp. Fig 1.**). A R^2 of 0.82 and a *p*-value less than 2.2 x 10-16 were found, indicating a very strong positive linear relationship between sensor and HPLC data.

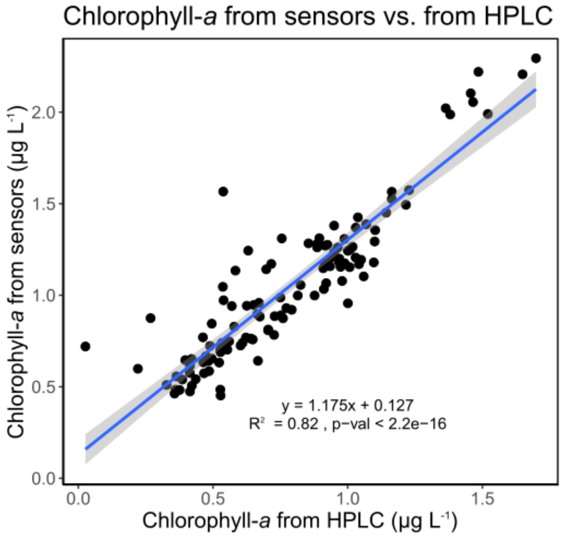

Supp. Figure 1. Ordinary least square linear relationship between high-frequency chl-*a* fluorescence from sensors averaged over a 1 hr period centered around 9:00 and discrete HPLC measurements of chl-*a* concentrations.

Similarly, we compared µ with the daily net change rate of chl-*a* based on daily HPLC measurements. To do so, we calculated the daily net change rate of chlorophyll-a based on daily HPLC measurements of chl-*a* (from d1 to d21 in all mesocosms), as $r_{t+1} = \ln\left(\frac{C_{t+1}}{C_t}\right)$, with r_{t+1} the daily net change of chl-*a* at day t+1, in d^{-1}, and C_{t+1} and C_t the HPLC-measured concentration of chl-*a* at day t+1 and t, respectively, in µg L^{-1}. We then compared these daily net change rates with the growth rates measured with the sensors. We performed an ordinary least square linear relationship and found a significant linear relationship (p-val = 8.146×10^{-5}) (**Supp. Fig 2.**).

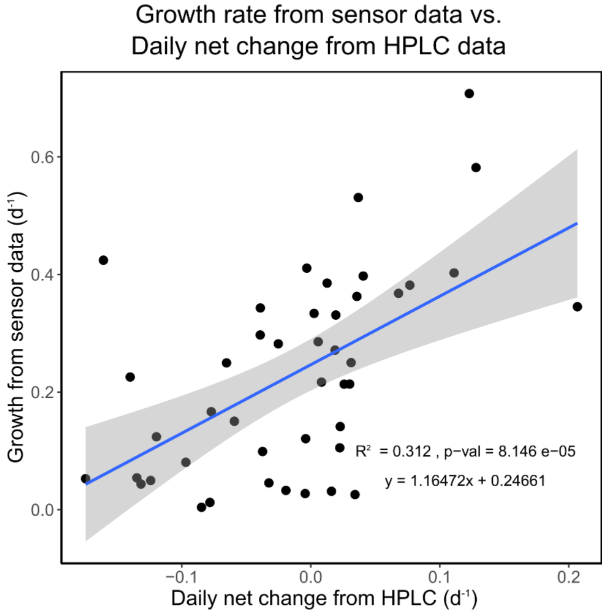

Supp. Figure 2. Ordinary least square linear relationship between phytoplankton growth rate estimated from sensor data and daily net change rate calculated from HPLC measurements of chl-*a* concentrations.

Gross Primary Production (GPP) and community Respiration (R) from high-frequency dissolved oxygen measurements

GPP and R were estimated from high-frequency dissolved oxygen data using a free-water diel oxygen method based on the classical technique from Odum (Odum 1956). Each dissolved oxygen cycle was separated in periods of positive instantaneous Net Community Production (NCP) and periods of negative instantaneous NCP (periods of increasing and decreasing dissolved oxygen concentration, respectively). For each positive and negative NCP periods, the dissolved oxygen data were smoothed with a 5-point sigmoidal model. The fundamental equation of the method is presented as **Eq. (S3)**:

$$\frac{\Delta O_2}{\Delta t} = GPP - R - F - A$$

The instantaneous change in dissolved oxygen $\frac{\Delta O2}{\Delta t}$ is considered to depend on GPP, R, and on F, which represents the physical oxygen exchange between the water and the atmosphere, and A, which encompasses all other phenomena which could affect the dissolved oxygen concentration. A was taken as null in the present work as in most other studies (Soulié *et al.* 2021). F was calculated as follows (**Eq. S4**):

$$F = (k \times (O_2 - O_{2sat}))/Z_{mix}$$

In this equation, k represents the piston velocity coefficient, O2 and O2sat the concentration and saturation of dissolved oxygen respectively, and Zmix the water column mixing depth, which is the mesocosm water column length in the case of mixed mesocosms. The k value was taken as k=0.000156 m min^{-1} from the literature (Alcaraz *et al.* 2001). Then, k was corrected for temperature and salinity using the high-frequency sensors data and following the procedure described in Soulié *et al*. (2021). Then, instantaneous NCP was calculated from the following equation (**Eq. S5**):

$$NCP(t) = O_2(t) - O_2(t-1) - F(t)$$

In this equation, O2(t) and O2(t − 1) are the dissolved oxygen concentration at time t and t − 1, respectively, and F(t) the exchange factor at time t. From this instantaneous NCP data, daily metabolic parameters were inferred. First, the respiration occurring during daylight, Rdaytime, was estimated with **Eq. (S6):**

$R_{daytime}$= (mean of NCP during a 1h period centered around the max. NCP of the Negative NCP period) × duration of Positive NCP period ×60

In this equation, Rdaytime is expressed in $gO_2\ m^{-3}\ d^{-1}$, the mean instantaneous NCP in $gO_2\ m^{-3}\ min^{-1}$, and the duration of the positive NCP period in h. The respiration occurring at night, Rnight, was estimated from **Eq.** (**S7**):

$$R_{night} = (\text{mean of NCP during the Negative NCP period}) \times \text{duration of Negative NCP period} \times 60$$

Similarly, Rnight is expressed in $gO_2\ m^{-3}\ d^{-1}$, the mean instantaneous NCP in $gO_2\ m^{-3}\ min^{-1}$, and the duration of the negative NCP period in h. Finally, daily R is the sum of Rdaytime and Rnight. Daily GPP is then calculated with the following equation **Eq. (S8)**:

$$GPP = R_{daytime} + (\text{mean of NCP during the Positive NCP period}) \times \text{duration of Positive NCP period} \times 60$$

Daily GPP and Rnight are expressed in $gO_2\ m^{-3}\ d^{-1}$, the mean instantaneous NCP in $gO_2\ m^{-3}\ min^{-1}$, and the duration of the positive NCP period in h. Daily NCP is then calculated as the difference between daily GPP and daily R.

Initial ratio matrix used for the CHEMTAX analyses

Supp. Table 1. Initial ratio matrix used for the CHEMTAX analyses. Chl-c3: Chlorophyll-c3; Peri: Peridinin; 19’-But-F: 19’-Butanoyloxyfucoxanthin; Fuco: Fucoxanthin; 19’-HF: 19’-Hexanoyloxyfucoxanthin; Zea: Zeaxanthin; Allo: Alloxanthin; Chl-b: Chlorophyll-b; Diadino: Diadinoxanthin; 4-Keto: 19’-Hexanoyloxy-4-ketofucoxanthin; Chl-a: Chlorophyll-a; Chloro: Chlorophytes; Crypto: Cryptophytes; Cyano: Cyanobacteria; Dino: Dinoflagellates; Hapto: Haptophytes.

	Chl-*c*3	Peri	19’-But-F	Fuco	19’-HF	Zea	Allo	Chl-*b*	Diadino	4-Keto	Chl-*a*
Chloro	0	0	0	0	0	0.00955	0	0.2627	0	0	1
Crypto	0	0	0	0	0	0	0.228	0	0	0	1
Cyano	0	0	0	0	0	0.348	0	0	0	0	1
Diatom	0.065	0	0	1.02	0	0	0	0	0.132	0	1
Dino1	0	1.06	0	0	0	0	0	0	0.239	0	1
Dino2	0.032	0	0.061	0.142	0.187	0	0	0	0	0	1
Hapto 6-8	0.047	0	0.246	0.585	0.538	0	0	0	0	0.065	1
Hapto 3-4	0.046	0	0	0	1.07	0	0	0	0.1011	0	1

Results of the statistical analyses

Supp. Table 2. Summary table of the p values and the F-values obtained with the RM-ANOVA (with treatment as fixed factor and time as random factor) comparing variables in the HW and in the control treatments over the entire experiment and over the HW1, HW2, Post-HW1 and Post-HW2 periods. Effect sizes expressed as the difference in % are also indicated. When the assumptions for a parametric

test were not met, despite transforming the data, a Kruskal–Wallis test was used instead and indicated by "K-W". P values lower than 0.05 were considered as significant and are indicated in bold in the table.

**Variable**	**Period**	**Test**	**P-value**	**F-value**	**diff(%)**
R	d1-d32	RM-ANOVA	**0.0396**	(1,11) 5.45	-1.74
R	d1-d32	RM-ANOVA	0.961	(1,31) 0.002	0.17
R	d1-d5	RM-ANOVA	**0.0048**	(1,4) 32.23	7.06
R	d6-d10	RM-ANOVA	**0.0421**	(1,4) 8.677	-4.4
R	d11-d15	RM-ANOVA	**0.0042**	(1,4) 34.621	7.01
R	d16-d20	RM-ANOVA	**0.0102**	(1,4) 20.907	-4.49
GPP	d1-d32	RM-ANOVA	0.0512	(1,31) 4.11	1.35
GPP	d21-d32	RM-ANOVA	0.3321	(1,11) 1.029	0.76
GPP	d1-d5	RM-ANOVA	**0.0084**	(1,4) 23.379	7.64
GPP	d6-d10	RM-ANOVA	0.6423	(1,4) 0.252	-0.64
GPP	d11-d15	RM-ANOVA	0.1887	(1,4) 2.5	0.83
GPP	d16-d20	RM-ANOVA	0.6245	(1,4) 0.28	-0.88
GPP:R Ratio	d1-d32	RM-ANOVA	0.0795	(1,31) 3.287	1.37
GPP:R Ratio	d1-d5	RM-ANOVA	0.6777	(1,4) 0.20	0.73
GPP:R Ratio	d6-d10	RM-ANOVA	**0.0262**	(1,4) 11.874	4.04
GPP:R Ratio	d11-d15	RM-ANOVA	**0.0041**	(1,4) 35.16	-5.62
GPP:R Ratio	d16-d20	RM-ANOVA	0.1194	(1,4) 3.903	3.91
GPP:R Ratio	d21-d32	RM-ANOVA	**0.0054**	(1,11) 11.920	2.32
GPPchla	d1-d32	RM-ANOVA	0.6613	(1,31) 0.195	-1.12
GPPchla	d1-d5	RM-ANOVA	**0.0371**	(1,4) 9.4567	-20.35
GPPchla	d6-d10	RM-ANOVA	0.7148	(1,4) 0.154	6.96
GPPchla	d11-d15	RM-ANOVA	**0.0017**	(1,4) 56.42	-14.52
GPPchla	d16-d20	RM-ANOVA	**0.0033**	(1,4) 39.50	-16.73
GPPchla	d21-d32	RM-ANOVA	0.3367	(1,11) 1.008	14.79
O_2	d1-d32	RM-ANOVA	**0.0001**	(1,30) 58.12	-3.22
O_2	d1-d5	RM-ANOVA	**0.0001**	(1,4) 617.66	-5.32
O_2	d6-d10	RM-ANOVA	**0.0001**	(1,4) 117.18	-2.82
O_2	d11-d15	RM-ANOVA	**0.0001**	(1,4) 209.26	-6.32
O_2	d16-d20	RM-ANOVA	**0.0001**	(1,4) 123.11	-3.44
O_2	d21-d32	RM-ANOVA	0.0702	(1,10) 4.11	-0.85
Chla_fluo	d1-d32	RM-ANOVA	0.6138	(1,30) 0.26	9.71
Chla_fluo	d1-d5	RM-ANOVA	**0.0308**	(1,4) 10.69	36.56
Chla_fluo	d6-d10	RM-ANOVA	0.8459	(1,4) 0.042	1.24
Chla_fluo	d11-d15	RM-ANOVA	**0.0003**	(1,4) 148.93	17.72
Chla_fluo	d16-d20	RM-ANOVA	**0.0002**	(1,4) 168.52	21.86
Chla_fluo	d21-d32	RM-ANOVA	0.6239	(1,12) 0.25	-8.35
µ	d1-d32	RM-ANOVA	0.5674	(1,31) 0.33	7.36
µ	d1-d5	RM-ANOVA	0.4766	(1,4) 0.6155	32.61
µ	d6-d10	RM-ANOVA	0.107	(1,4) 4.2934	-47.75
µ	d11-d15	RM-ANOVA	0.0684	(1,4) 6.13657	79.73
µ	d16-d20	RM-ANOVA	0.9387	(1,4) 0.0067	1.43
µ	d21-d32	RM-ANOVA	0.7727	(1,11) 0.087671	6.8
L	d1-d32	RM-ANOVA	0.2007	(1,31) 1.70918	15.08
L	d1-d5	RM-ANOVA	0.476	(1,4) 0.61716	18.93

L	d6-d10	RM-ANOVA	0.4548	(1,4) 0.6837	-33.63
L	d11-d15	RM-ANOVA	0.109	(1,4) 4.224	70.84
L	d16-d20	RM-ANOVA	0.6168	(1,4) 0,2934	13.76
L	d21-d32	RM-ANOVA	0.264	(1,11) 1.38533	21.94
µ:L	d1-d32	K-W	0.3735		-1.17
µ:L	d1-d5	K-W	0.2506		0.91
µ:L	d6-d10	RM-ANOVA	0.4883	(1,4) 0.581	-1.13
µ:L	d11-d15	RM-ANOVA	0.0655	(1,4) 6.3386	8.81
µ:L	d16-d20	K-W	0.3472	(1,4) 0.88364	-10.88
µ:L	d21-d32	RM-ANOVA	0.264	(1,11) 1.38533	-1.83
NH_4^+	d1-d32	RM-ANOVA	**0.0096**	(1,12) 9.4571	113.65
NH_4^+	d1-d5	RM-ANOVA	0.2055	(1,2) 3.425	17.91
NH_4^+	d6-d10	RM-ANOVA	0.1795	(1,2) 4.12	-11.84
NH_4^+	d11-d15	RM-ANOVA	0.1594	(1,2) 4.81	93.79
NH_4^+	d16-d21	RM-ANOVA	**0.0017**	(1,3) 115.74	291.43
NO_2^-	d1-d32	RM-ANOVA	**0.0036**	(1,12) 12.99	15.46
NO_2^-	d1-d5	RM-ANOVA	0.0765	(1,2) 11.589	6.95
NO_2^-	d6-d10	RM-ANOVA	0.2831	(1,2) 2.115	7.22
NO_2^-	d11-d15	RM-ANOVA	0.1057	(1,2) 7.9917	3.63
NO_2^-	d16-d21	RM-ANOVA	**0.0012**	(1,3) 146.13	37.52
NO_3^-	d1-d32	RM-ANOVA	0.7457	(1,12) 0.1102	3.02
NO_3^-	d1-d5	RM-ANOVA	0.0587	(1,2) 15.5519	-16.42
NO_3^-	d6-d10	RM-ANOVA	0.6084	(1,2) 0.36231	-18.17
NO_3^-	d11-d15	RM-ANOVA	0.6142	(1,2) 0.3496	5.28
NO_3^-	d16-d21	RM-ANOVA	**0.0223**	(1,3) 19.0122	36.94
PO_4^{3-}	d1-d32	RM-ANOVA	**0.0058**	(1,12) 11.184	6.99
PO_4^{3-}	d1-d5	RM-ANOVA	0.0908	(1,2) 9.5328	11.31
PO_4^{3-}	d6-d10	RM-ANOVA	**0.0368**	(1,2) 25.67	10.29
PO_4^{3-}	d11-d15	RM-ANOVA	0.8334	(1,2) 0.057	0.93
PO_4^{3-}	d16-d21	RM-ANOVA	0.3087	(1,3) 1.4954	5.40
SiO_2	d1-d32	RM-ANOVA	0.0658	(1,12) 4.0982	13.34
SiO_2	d1-d5	RM-ANOVA	0.2837	(1,2) 2.1071	10.14
SiO_2	d6-d10	RM-ANOVA	**0.001**	(1,2) 978.95	41.49
SiO_2	d11-d15	RM-ANOVA	**0.0002**	(1,2) 4103.6	26.83
SiO_2	d16-d21	RM-ANOVA	0.5642	(1,3) 0.4176	-8.72
NP_ratio	d1-d32	RM-ANOVA	0.3616	(1,12) 0.8997	23.77
NP_ratio	d1-d5	RM-ANOVA	0.0699	(1,2) 12.83117	-17.29
NP_ratio	d6-d10	RM-ANOVA	0.3963	(1,2) 1.147	-22.97
NP_ratio	d11-d15	RM-ANOVA	**0.0183**	(1,2) 53.0949	24.32
NP_ratio	d16-d21	RM-ANOVA	**0.0264**	(1,3) 16.715	86.34
chla_tot	d1-d32	RM-ANOVA	0.2833	(1,12) 1.261	9.64
chla_tot	d1-d5	RM-ANOVA	0.5502	(1,2) 0.507	16.58
chla_tot	d6-d10	RM-ANOVA	0.5417	(1,2) 0.532	13.55
chla_tot	d11-d15	RM-ANOVA	0.86	(1,2) 0.04	1.26
chla_tot	d16-d21	RM-ANOVA	0.8688	(1,3) 0.032	1.96
chla_inf_3	d1-d32	RM-ANOVA	**0.007**	(1,12) 10.53	56.48
chla_inf_3	d1-d5	RM-ANOVA	**0.0395**	(1,2) 23.8384	44.76
chla_inf_3	d6-d10	RM-ANOVA	0.2751	(1,2) 2.215	13.19

chla_inf_3	d11-d15	RM-ANOVA	0.1413	(1,2) 5.617	154.22
chla_inf_3	d16-d21	RM-ANOVA	0.3554	(1,3) 1.189	56.67
chla_sup_3_inf_20	d1-d32	RM-ANOVA	0.657	(1,12) 0.207	-4.41
chla_sup_3_inf_20	d1-d5	RM-ANOVA	0.3915	(1,2) 1.176	-15.79
chla_sup_3_inf_20	d6-d10	RM-ANOVA	0.5881	(1,2) 0.407	13.18
chla_sup_3_inf_20	d11-d15	RM-ANOVA	0.2271	(1,2) 2.967	-32.35
chla_sup_3_inf_20	d16-d21	RM-ANOVA	0.514	(1,3) 0.544	14.14
chla_sup_20	d1-d32	K-W	0.8663		26.07
chla_sup_20	d1-d5	RM-ANOVA	0.6028	(1,2) 0.374	60.79
chla_sup_20	d6-d10	RM-ANOVA	0.4226	(1,2) 1	-71.43
chla_sup_20	d11-d15	RM-ANOVA	0.438	(1,2) 0.923	144
chla_sup_20	d16-d21	RM-ANOVA	0.3011	(1,3) 1.55	-57.73
DLI	d1-d32	RM-ANOVA	**0.0049**	(1,32) 9.1267	11.30
DLI	d1-d5	RM-ANOVA	0.9821	(1,4) 0.0006	0.06
DLI	d6-d10	RM-ANOVA	0.6487	(1,4) 0.2418	1.14
DLI	d11-d15	K-W	0.9168		-4.32
DLI	d16-d20	RM-ANOVA	0.3056	(1,4) 1.3779	-4.88
DLI	d21-d32	RM-ANOVA	**0.0001**	(1,11) 34.64	34.01
Chlorophytes	d1-d21	K-W	**0.0001**		514
Chlorophytes	d1-d5	RM-ANOVA	**0.0026**	(1,4) 44.69	-20.75
Chlorophytes	d6-d10	RM-ANOVA	0.9173	(1,4) 0.01	8.86
Chlorophytes	d11-d15	RM-ANOVA	**0.0119**	(1,4) 19.2	*n.a.*
Chlorophytes	d16-d21	K-W	**0.0021**		*n.a.*
Cyanobacteria	d1-d21	K-W	**0.0065**		90.54
Cyanobacteria	d1-d5	RM-ANOVA	0.1251	(1,4) 3.74	-13.87
Cyanobacteria	d6-d10	RM-ANOVA	**0.0072**	(1,4) 25.56	*n.a.*
Cyanobacteria	d11-d15	RM-ANOVA	0.0744	(1,4) 5.76	1291
Cyanobacteria	d16-d21	RM-ANOVA	**0.0295**	(1,5) 9.10	1091
Diatoms	d1-d21	K-W	0.2017		2.15
Diatoms	d1-d5	RM-ANOVA	0.1413	(1,4) 3.346	-12.62
Diatoms	d6-d10	RM-ANOVA	0.7896	(1,4) 0.081	-10.35
Diatoms	d11-d15	RM-ANOVA	0.0609	(1,4) 6.69	1037.8
Diatoms	d16-d21	K-W	**0.0028**		7636
Dinoflagellates	d1-d21	RM-ANOVA	0.4165	(1,20) 0.688	7.93
Dinoflagellates	d1-d5	RM-ANOVA	0.0996	(1,4) 4.561	-18.59
Dinoflagellates	d6-d10	K-W	0.1745		39.04
Dinoflagellates	d11-d15	RM-ANOVA	0.6393	(1,4) 0.2563	-9.26
Dinoflagellates	d16-d21	RM-ANOVA	0.0653	(1,5) 5.538	29.18
Hapto 6-8	d1-d21	RM-ANOVA	**0.0001**	(1,20) 31.49	21.37
Hapto 6-8	d1-d5	RM-ANOVA	0.2074	(1,4) 2.256	13.99
Hapto 6-8	d6-d10	RM-ANOVA	**0.0437**	(1,4) 7.346	18.62
Hapto 6-8	d11-d15	RM-ANOVA	**0.0159**	(1,4) 16.11	28.82
Hapto 6-8	d16-d21	RM-ANOVA	**0.0046**	(1,5) 23.79	22.26
Hapto 3-4	d1-d21	RM-ANOVA	**0.0006**	(1,20) 16.59	-20.69
Hapto 3-4	d1-d5	RM-ANOVA	0.1219	(1,4) 2.26	14.32
Hapto 3-4	d6-d10	RM-ANOVA	0.1007	(1,4) 4.52	-19.92
Hapto 3-4	d11-d15	RM-ANOVA	**0.0371**	(1,4) 9.45	-28.28
Hapto 3-4	d16-d21	RM-ANOVA	**0.0001**	(1,5) 55.99	-27.86

Supp. Table 3. Summary table of the p values obtained with the Kruskal–Wallis test comparing daily variables in the HW and in the control treatments. Effect sizes expressed as the difference in % are also indicated. P values lower than 0.05 were considered as significant and are indicated in bold in the table.

Day of experiment	GPP	R	GPP:R_ratio	GPPchla	µ	L	µ:L_ratio
1	0.8273	0.1266	**0.0495**	**0.0495**	0.5127	0.5127	0.1266
2	**0.0495**	**0.0495**	0.1266	0.2752	0.1266	0.2752	**0.0495**
3	**0.0495**	0.1266	0.5127	**0.0495**	**0.0495**	**0.0495**	**0.0495**
4	**0.0495**	0.1266	0.5127	**0.0495**	**0.0495**	**0.0495**	0.8273
5	**0.0495**	0.2752	0.8273	**0.0495**	**0.0495**	**0.0495**	**0.0495**
6	**0.0495**	0.5127	0.8273	**0.0495**	0.8273	0.5127	0.1266
7	0.2752	**0.0495**	**0.0495**	0.5127	**0.0495**	**0.0495**	0.8273
8	0.2752	0.5127	0.5127	0.1266	**0.0495**	**0.0495**	0,2752
9	0.2752	**0.0495**	0.1266	**0.0495**	0.1266	**0.0495**	0.5127
10	0.2752	**0.0495**	**0.0495**	0.5127	**0.0495**	**0.0495**	0.5127
11	0.8273	0.8273	0.8273	0.5127	0.8273	0.8273	0.2752
12	0.8273	**0.0495**	**0.0495**	0.2752	0.8273	0.8273	0.5127
13	0.1266	**0.0495**	0.1266	0.1266	0.1266	0.1266	0.1266
14	0.8273	**0.0495**	**0.0495**	0.1266	**0.0495**	**0.0495**	0.2752
15	0.8273	**0.0495**	0.1266	0.2752	0.5127	0.5127	**0.0495**
16	**0.0495**	**0.0495**	0.1266	**0.0495**	0.2752	0.2752	0.5127
17	1.0000	0.0833	0.0833	0.5637	0.8273	0.5127	0.5127
18	0.0833	1.0000	1.0000	0.0833	0.2482	0.5637	0.5637
19	1.0000	0.0833	0.0833	0.5637	0.0833	0.0833	0.2482
20	0.0833	0.5637	0.0833	0.5637	0.0833	0.0833	0.0833
21	0.0833	0.5637	0.5637	0.5637	0.2482	0.2482	0.0833
22	0.5637	0.0833	**0.0433**	0.0833	0.0833	0.5637	0.0833
23	1.0000	0.0833	0.2482	0.0833	0.5637	0.5637	0.2482
24	0.0833	1.0000	1.0000	0.0833	0.5637	0.5637	0.5637
25	0.0833	1.0000	1.0000	0.0833	0.5637	0.2482	0.0833
26	1.0000	0.2482	0.5637	0.2482	0.0833	0.0833	0.0833
27	1.0000	0.5637	0.5637	1.0000	0.5637	0.5637	0.5637
28	1.0000	0.5637	0.5637	0.5637	1.0000	1.0000	1.0000
29	0.5637	0.5637	1.0000	0.5637	1.0000	0.2482	0.5637
30	1.0000	0.2482	0.0833	0.2482	0.2482	1.0000	0.0833
31	0.5637	0.5637	0.5637	**0.0495**	**0.0495**	**0.0495**	**0.0495**
32	0.2482	0.5637	0.5637	**0.0495**	**0.0495**	**0.0495**	**0.0495**

Supp. Table 3. Continued.

Day of experiment	SiO_2	NO_2^-	NO_3^-	PO_4^{3-}	NH_4^+	NP_ratio
1	0.8273	0.0765	**0.0495**	0.5127	0.1840	0.5127
2						
3	0.8273	**0.0495**	**0.0495**	**0.0463**	**0.0463**	**0.0495**
4						
5	**0.0495**	0.2752	**0.0495**	**0.0463**	0.2752	**0.0495**
6	**0.0463**	**0.0495**	**0.0495**	**0.0495**	0.2683	0.5127
7	**0.0463**	0.2752	**0.0495**	**0.0463**	**0.0463**	**0.0495**
8						
9	**0.0495**	0.8248	**0.0495**	**0.0463**	**0.0463**	**0.0495**
10						
11	**0.0463**	0.3687	**0.0495**	**0.0463**	**0.0495**	**0.0495**
12	**0.0495**	0.5127	0.1840	0.8273	**0.0495**	**0.0495**
13						
14	**0.0495**	0.2752	0.2752	**0.0495**	**0.0495**	**0.0495**
15						
16	**0.0495**	**0.0495**	**0.0463**	0.6579	**0.0463**	**0.0495**
17	**0.0495**	**0.0495**	**0.0463**	0.8222	**0.0463**	**0.0495**
18						
19	**0.0495**	**0.0495**	**0.0495**	0.1840	**0.0463**	**0.0495**
20						
21	**0.0495**	**0.0495**	**0.0495**	**0.0495**	**0.0495**	**0.0495**

Supp. Table 3. Continued.

Day of experiment	Chlorophytes	Cyano	Diatoms	Dinoflagellates	Hapto 6-8	Hapto 3-4
1	0.2752	0.5126	0.5126	0.5126	0.1266	0.5126
2	**0.0495**	0.8272	0.2752	0.2752	0.2752	0.1266
3	0.1266	**0.0495**	0.8272	**0.0495**	**0.0495**	0.5126
4	0.1266	0.5126	**0.0495**	**0.0495**	**0.0495**	**0.0495**
5	0.1266	*n.a.*	**0.0495**	**0.0495**	**0.0495**	**0.0495**
6	**0.0495**	**0.0369**	**0.0495**	0.5126	**0.0495**	0.2752
7	0.2463	**0.0369**	**0.0495**	0.8272	**0.0495**	**0.0495**
8	*n.a*	**0.0369**	0.5126	0.5126	**0.0495**	**0.0495**
9	0.1213	**0.0369**	**0.0495**	0.5126	0.1266	**0.0495**
10	**0.0369**	**0.0369**	**0.0463**	**0.0495**	0.5126	**0.0495**
11	0.0528	**0.0528**	0.0832	0.0832	0.0832	0.0832
12	0.0528	0.0528	0.0528	0.0832	0.0832	0.0832
13	0.0528	0.0755	0.0528	0.2482	0.2482	0.0832
14	0.0528	0.5186	*n.a.*	0.0832	0.5637	0.2482
15	0.0528	0.4142	0.5186	0.0832	1.0000	1.0000
16	0.0528	0.4142	0.2206	1.0000	0.5637	0.0832
17	0.0528	0.2206	0.2206	0.0832	0.0832	0.0832
18	0.0528	0.0755	0.5186	1.0000	0.0832	0.0832
19	0.0528	0.0755	0.2206	0.0832	0.5637	0.0832
20	0.0528	0.0528	0.2206	0.0832	0.0832	0.0832
21	0.0528	0.0755	0.0528	0.0832	0.0832	0.0832

References

Alcaraz, M., C. Marrasé, F. Peters, L. Arin, and A. Malits. 2001. Seawater-atmosphere O2 exchange rates in open-top laboratory microcosms: application for continuous estimates of planktonic primary production and respiration. *J. Exp. Mar. Biol. Ecol*. **257**:1-12. doi:10.1016/S0022-0981(00)00328-2

Neveux, J., C. Dupouy, J. Blanchot, A. Le Bouteiller, M. R. Landry, and S. L. Brown. 2003. Diel dynamics of chlorophylls in high-nutrient, low chlorophyll waters of the equatorial Pacific (180°): Interactions of growth, grazing, physiological responses, and mixing. *J. Geophys. Res*. **108**(C12):8140. Doi: 10.1029/2000JC000747

Ritchie, R. J. 2006. Consistent sets of spectrophotometry chlorophyll equations for acetone, methanol, and ethanol solvents. *Photosynth. Res*. **89**: 27-41. Doi: 10.1007/s11120-006-9065-9

Soulié, T., S. Mas, D. Parin, F. Vidussi, and B. Mostajir. 2021. A new method to estimate planktonic oxygen metabolism using high-frequency sensor measurements in mesocosm experiments and considering daytime and nighttime respirations. *Limnol. Oceanogr. Methods*. **19**: 303-316. Doi: 10.1002/lom3.10424

Soulié, T., F. Vidussi, J. Courboulès, S. Mas, and B. Mostajir. 2022. Metabolic responses of plankton to warming during different productive seasons in coastal Mediterranean waters revealed by in situ mesocosm experiments. *Sci. Rep*. **12**:9001. Doi: 10.1038/s41598-022-12744-x
